# Impact of Enhanced Phagocytosis of Glycated Erythrocytes on Human Endothelial Cell Functions

**DOI:** 10.3390/cells11142200

**Published:** 2022-07-14

**Authors:** Chloé Turpin, Marie Laurine Apalama, Bastian Carnero, Alberto Otero-Cacho, Alberto P. Munuzuri, Maria Teresa Flores-Arias, Erick Vélia, Olivier Meilhac, Emmanuel Bourdon, Ezequiel Álvarez, Philippe Rondeau

**Affiliations:** 1 UMR 1188 Diabète Athérothombose Thérapies Réunion Océan Indien (DéTROI), INSERM, Université de La Réunion, 97400 Saint-Denis, France; chloe.turpin@univ-reunion.fr (C.T.); marie.apalama@gmail.com (M.L.A.); olivier.meilhac@inserm.fr (O.M.); 2 Photonics4Life Research Group, Applied Physics Department, Faculty of Physics, Institute of Materials (iMATUS), Universidade de Santiago de Compostela, 15782 Santiago de Compostela, Spain; bastian.carnero.groba@usc.es (B.C.); flores@usc.es (M.T.F.-A.); 3 BFlow S.L., Edificio Emprendia, 15782 Santiago de Compostela, Spain; 4 Galician Center for Mathematical Research and Technology (CITMAga) and Group of Nonlinear Physics, Department of Physics, Universidade de Santiago de Compostela, 15782 Santiago de Compostela, Spain; alberto.otero.cacho@usc.es (A.O.-C.); alberto.perez.munuzuri@usc.es (A.P.M.); 5 FlowReserve Labs S.L., Edificio Emprendia, 15706 Santiago de Compostela, Spain; 6 Clinique Sainte-Clotilde, Groupe Clinifutur, Pôle Mère Enfant, 97490 Sainte-Clotilde, France; erick.velia@clinifutur.net; 7 Centre Hospitalier Universitaire de La Réunion, 97400 Saint Denis, France; 8 Cardiology Group, Health Research Institute of Santiago de Compostela (IDIS), Hospital Universitario de Santiago de Compostela (SERGAS), Trav. Choupana s/n, 15706 Santiago de Compostela, Spain; ezequiel.alvarez.castro@gmail.com; 9 Centro de Investigación Biomedica en Red de Enfermedades Cardiovasculares (CIBERCV), 28029 Madrid, Spain; 10 Departamento de Farmacología, Farmacia y Tecnología Farmacéutica, Universidade de Santiago de Compostela, 15782 Santiago de Compostela, Spain

**Keywords:** diabetes, glycation, erythrocytes, erythrophagocytosis, endothelial cells, red blood cells, oxidative stress

## Abstract

Diabetes is associated with a high mortality rate due to vascular complications. Chronic hyperglycemia in diabetes leads to enhanced oxidative stress and glycation. Here, we explored the impact of glycation on human erythrocyte characteristics and capacity to affect endothelial cell function following erythrophagocytosis. Native and glucose-mediated glycated erythrocytes were prepared and characterized in terms of structural and deformability modifications. Erythrocyte preparations were tested for their binding and phagocytosis capacity as well as the potential functional consequences on human endothelial cell lines and primary cultures. Oxidative modifications were found to be enhanced in glycated erythrocytes after determination of their deformability, advanced glycation end-product content and eryptosis. Erythrophagocytosis by endothelial cells was significantly increased when incubated in the presence of glycated erythrocytes. In addition, higher iron accumulation, oxidative stress and impaired endothelial cell permeability were evidenced in cells previously incubated with glycated erythrocytes. When cultured under flow conditions, cellular integrity was disrupted by glycated erythrocytes at microvessel bifurcations, areas particularly prone to vascular complications. This study provides important new data on the impact of glycation on the structure of erythrocytes and their ability to alter endothelial cell function. Increased erythrophagocytosis may have a deleterious impact on endothelial cell function with adverse consequences on diabetic vascular complications.

## 1. Introduction

Diabetes constitutes an increasingly prevalent pathology affecting more than 5 percent of the world’s total population [1]. This pathology is closely associated with vascular dysfunctions and atherosclerotic cardiovascular disease (ACVD), which represents the leading cause of mortality of diabetic persons [2,3]. Mortality due to heart attacks and strokes is increased two- to three-fold in diabetic persons [4]. Enhanced ACVD occurrence in diabetes is due to microvascular and macrovascular complications such as coronary heart disease and ischemic strokes [5]. Although the specific mechanisms underlying ACVD in diabetes remain unknown, research studies are increasingly identifying the vascular endothelium as a key target of circulating stressors [6].

Erythrocytes represent vital abundant circulating elements that may be transformed into active stressors toward the vascular endothelium in ACVD [7]. A direct relationship was established between the erythrocyte shape (width) and coronary artery disease rate [8]. In ACVD, enhanced neovascularization of atherosclerotic plaque suggested an intense participation of erythrocytes in the progression of the pathology [9]. More recently, a higher susceptibility for intraplaque hemorrhages has characterized atherosclerotic lesions issued from diabetics versus non-diabetics [10].

Endothelium barrier and microvessel disruption may bring large amounts of erythrocytes into the plaque, participating in its progression and complication [11]. Then, extravasated erythrocytes, as a source of cholesterol, hemoglobin and iron, can generate a deleterious environment contributing to oxidative stress and plaque rupture [11,12,13].

Oxidative stress (OS) and damage can also occur in the vascular compartment and imply impaired/oxidized erythrocytes as central stressors in the promotion of diabetic complications [3,12,14]. In hyperglycemic conditions, erythrocytes are subject to glycation. This glycation phenomenon leads to the occurrence of deleterious compounds termed advanced glycation end-products (AGEs) associated with diabetic vascular complications [14]. Circulating AGEs can interact with vascular cells through a specific receptor family named RAGE. Very recently, our group identified the impaired structure of erythrocytes following glycation associated with an enhanced capacity to interact with and be phagocytosed by endothelial cells [15,16].

If accumulating evidence implicates erythrocytes as active contributors in diabetes disorder development and vascular complications, too few data concern glycated erythrocyte impact on endothelial cell functioning via phagocytosis. Hence, we hypothesized that glycation may induce damage in erythrocytes, rendering them more prone to altering endothelial cell functioning.

## 2. Materials and Methods

### 2.1. Reagents

Bicinchoninic acid (BCA), D-glucose, Drabkin’s reagent, ferrous sulfate (FeSO_4_), fluorescein isothiocyanate-dextran (FITCDextran), formic acid, mannitol and PKH67 (green fluorescent cell linker) were all purchased from Sigma-Aldrich (St. Louis, MO, USA). Fetal bovine serum (FBS), L-glutamine, penicillin/streptomycin, amphotericin B and HAT (hypoxanthine/aminopterin/thymidine) were obtained from Pan Biotech (Aidenbach, Germany). 3-(2-Pyridyl)-5,6-diphenyl-1,2,4-triazine-4′,4′′-disulfonic acid sodium salt (ferrozine) and 2,9-Dimethyl-1,10-phenanthroline(neocuproine) were purchased from Alfa Aesar (Kandel, Germany). MitoSOX Red mitochondrial superoxide indicator and pHrodo Red were purchased from Thermo Fisher (Carlsbad, CA, USA).

### 2.2. Erythrocyte Preparations

Erythrocytes were obtained from the French blood national agency (EFS-LR agreement number # 2018001378). Blood was collected in EDTA tubes (BD vacutainer^®^, Franklin Lakes, NJ, USA). Erythrocytes were washed 3 times with an isotonic Ringer-lactate solution (B. Braun, Melsungen, Germany) and prepared at 20% hematocrit in phosphate buffered saline solution/5 mM glucose (PBS/0.1% glucose). Glycation was induced by incubating erythrocytes in the absence (G0) or presence of D-glucose at three increasing concentrations (5 (G5), 50 (G50) and 100 mM (G100)). A previous study, performed in our laboratory, showed that such in vitro conditions represent a suitable erythrocyte glycation model, inducing HbA1c percentages similar to those that can be measured in diabetic patients [16]. After 5 days of incubation at 37 °C, erythrocytes were washed 3 times with Ringer-lactate solution before a direct use by FACS, ektacytometry and for endothelial cells stimulation. Quantitative determination of hemoglobin in erythrocytes was performed with Drabkin’s colorimetric assay.

### 2.3. Erythrocyte Senescence Characterization

Erythrocyte shape, AGE level, CD47 expression and eryptosis were measured by flow cytometry using Beckman Coulter CytoFLEX and Cytexpert software (v2.1, Beckman Coulter, Brea, CA, USA). A specific erythrocyte cell population was selected by gating and could be characterized by its typical location in a forward scatter (FSC) versus a side scatter (SSC) parameter graph.

For AGE level determination, washed erythrocytes were incubated with AGE antibody (Abcam, 1:200 dilution) for 1 h, followed by an incubation with anti-rabbit Alexa 647-conjugated secondary IgG (Thermo Fisher, 1:200 dilution).

For conformational changes of CD47 on erythrocytes membrane, washed samples were double stained with two distinct CD47 antibodies (10 μg/mL in HEPES) directed against two different epitopes: an APC-conjugated conformation-independent antibody (clone B6H12) and a FITC-conjugated conformation-dependent (clone 2D3) antibody (Thermo Fisher, 1:200 dilution).

For phosphatidylserine (PS) exposure determination, 100 μL of erythrocytes (1:50 dilution) was incubated for 30 min at RT with 2 μg/mL annexin V-FITC in the commercial buffer (BioLegend, San Diego, CA, USA) before flow cytometry analysis. Annexin V protein, which exhibits a high affinity for PS, was measured with an excitation wavelength of 488 nm and an emission wavelength of 530 nm.

### 2.4. Shear Stress Gradient Ektacytometry

The determination of erythrocyte membrane deformability was performed using the deformability module of the Laser-assisted Optical Rotational Deformability Cell Analyser (LoRRca MaxSis. RR Mechatronics, Zwaag, The Netherlands), as previously described [17]. The maximum elongation index (EI_max_) and the shear stress at half maximal deformation (SS_1/2_) can be obtained from deformability curves. Both parameters appeared to be relevant indicators of erythrocyte deformability capacity [18].

### 2.5. Endothelial Cell Culture

EA.hy926 endothelial cell line was obtained from the American Type Culture Collection (ATCC, USA, CRL-2922™) and cultured in Dulbecco’s Modified Eagle Medium (DMEM; Pan Biotech, Aidenbach, Germany) supplemented with 25 mM glucose, 10% heat-inactivated fetal bovine serum (FBS), 2 mM L-glutamine, 100 units/mL penicillin, 100 μg/mL streptomycin, 250 μg/mL amphotericin B and HAT (100 μmol/L hypoxanthine, 0.4 μmol/L aminopterin and 16 μmol/L thymidine). Cells were maintained at 37 °C with 5% CO_2_ in a humidified atmosphere.

Human umbilical vein endothelial cells (HUVECs) were obtained from human umbilical cords of patients who delivered babies at Sainte-Clotilde Clinic (Saint-Denis de La Réunion, France), with informed consent following the method previously described [19]. All procedures were approved according to French Law L.1243-3 modified by articles R1243-49 to 56, requiring the declaration of “Biobanking and preparation of cells and tissues from human body for research purpose” to MESR (French higher education and research ministry), Inserm (French National Institute for Health and Medical Research) and ANSM (French National Agency for Medicines and Health Products Safety) with the following references: C19-23 (Inserm), 2019-A01137-50 (IDRCB), and DC-2016-2614 (MESR). HUVECs were cultured on 0.2% (*w*/*v*) gelatin (Sigma-Aldrich) pre-coated flasks, grown in complete endothelial cell growth medium ENDOPAN-3 (Pan Biotech, Aidenbach, Germany) at 37 °C in 5% CO_2_ and maintained using standard cell culture.

For the flow assay (performed in Spain), HUVECs were isolated from freshly obtained human umbilical cords following the method previously described [20]. Umbilical cords were donated under written informed consent from mothers. All the procedures were approved by the Ethics Committee for Clinical Research at Galicia (Spain), according to the World Medical Association Declaration of Helsinki (Internal code 2013/326). Briefly, HUVECs were cultured on 0.2% (*w*/*v*) gelatin (Sigma-Aldrich; Merck Life Science S.L.U., Madrid, Spain) pre-coated flasks or dishes (Corning, New York, NY, USA) and grown in complete EGM-2 media (Endothelial Growth Medium-2, Lonza, Basel, Switzerland), containing 2% FBS between other components, in a humidity-saturated atmosphere with 5% CO_2_ at 37 °C. Cells for the experiments were used between the second and seventh passages.

Monkey kidney normal Vero cells (ATCC, CCL-81), used for only erythrophagocytosis assay, were cultured at 37 °C under a 5% CO_2_ atmosphere in complete Dulbecco’s Modified Eagle Medium (DMEM) (same medium composition as that used for EA.hy926 cells).

### 2.6. Real-Time Monitoring of HUVECs and EA.hy926 Cells

HUVECs or EA.hy926 cells were seeded at a concentration of 25,000 cells/well on xCELLigence 16-well E-plates (Acea Biosciences, San Diego, CA, USA) (gelatin-coated wells for HUVECs). The cell index was recorded continuously during cell growth until confluence, when a plateau was reached (until 90 h); cells were stimulated with erythrocyte samples at a density of 18.6 × 10^6^ cell/cm^2^. Cell index measurements were automatically collected every 15 min from 0 to 160 h.

### 2.7. Necrosis and Apoptosis Assay

For apoptosis/necrosis study, HUVECs were collected by trypsinization and washed with PBS after 6 h stimulation with erythrocytes. Then, cells were successively incubated with 2 μg/mL annexin V-FITC and 2 μg/mL propidium iodide (Sigma) in 100 μL binding buffer (BioLegend) for 30 min at RT before analysis by cytometry.

### 2.8. Protein Extraction from HUVECs and EA.hy926 Cells

For total iron and heme determination in HUVECs and EA.hy926, 6-well plates were used for cell culture. After 6 h or 12 h stimulation with erythrocyte preparations, cells were washed 3 times with PBS and non-specifically bound erythrocytes were lysed with distilled water. Cells were then detached by trypsin and lysed in 50 mM Tris buffer (pH 7.5), 0.1 mM EDTA and 8 M urea and total cell proteins were quantified by BCA assay.

### 2.9. Erythrophagocytosis Assays Using pHrodo and PKH67 Probes

One of the common ways to assess erythrophagocytosis consists of erythrocyte labeling with fluorescent probes such as pHrodo or PKH67 (Turpin, 2022 unpublished manuscript). Erythrocyte samples are resuspended at 20% hematocrit and labeled with the fluorescent markers pHrodo or PKH67, according to the manufacturer’s instructions. For pHrodo, 109 erythrocytes were labeled with 2 µL pHrodo Red succinimidyl ester in a final volume of 1 mL PBS for 60 min at 37 °C. For PKH67, 50 µL of erythrocytes (corresponding to 2.5 × 10^7^ cells) resuspended in 1 mL diluent buffer (supplied in the kit), were mixed to 2 μM PKH67 solution. The mixture was incubated at 37 °C for 10 min. Probe in excess was removed by several washing steps with PBS followed with 10 min centrifugation at 2000× *g*. Labeled erythrocytes were suspended with PBS at a final 20% of hematocrit prior to direct incubation with cultured endothelial cells for 6 h or 12 h. Endothelial cells treated in the same manner, but with unlabeled erythrocytes, were used as negative controls. After incubation and an intensive cell washing (three times with PBS) to get rid of non-phagocytosed erythrocytes, cells were subjected to flow-cytometry analysis, epi-fluorescence microscopy (Nikon Eclipse 80i) or confocal microscopy (Nikon Eclipse Ti2).

### 2.10. Human Umbilical Artery—Histological Analysis

From both umbilical cord arteries of approximately 6 cm in length, residual blood was removed by PBS flushing steps using a syringe equipped with a (23G) needle. Cleaned endothelial veins were filled either with glycated erythrocytes (G50 at a 20% hematocrit) or PBS (control) and sealed at each end. The cords were then fully immersed in PBS/ethanol solution (50:50, *v*/*v*) and incubated at 37 °C for 3 days. Following incubation, umbilical arteries were intensively washed with 4–5 volumes of PBS. After Wharton’s jelly removal, arteries were fixed in 4% paraformaldehyde and then embedded in OCT (Tissue Tek, Sakura Finetek, Flemingweg, The Netherlands) following standard histological protocols. Six micrometer-thick sections were prepared using a cryotome (Shandon™ cryotome FE, Thermo Scientific, Waltham, MA, USA) and were washed twice in PBS/0.1% Tween 20 before being blocked in PBS/0.1% Tween 20 containing 2% BSA. Next, sections were incubated with the following primary antibodies: anti-glycophorin A at 2.6 μg/mL (Dako, 1:500 dilution) combined with anti-PECAM (CD31) (Sigma, 1:500 dilution) or anti-von Willebrand factor at 3.17 μg/mL (Dako, 1:500 dilution) overnight at 4 °C. After two washing steps with PBS/0.1% Tween 20, sections were incubated with DAPI (4′,6′-diamidino-2-phenylindole) combined with secondary antibodies: donkey anti-rabbit Alexa Fluor 488 (Abcam, 1:500 dilution) for glycophorin A and von Willebrand factor and goat anti-mouse Alexa Fluor 594 (Abcam, 1:500 dilution) for CD31. After 2 h incubation at room temperature, sections were rinsed and the slides were mounted in Fluoromount and microscopy analysis was performed with a NanoZoomer S60 digital slide scanner (Hamamatsu).

### 2.11. Ferrozine Assay (Iron Level)

The quantitation of iron in endothelial cultured cells and in erythrocytes was performed by using the ferrozine-based colorimetric assay [21]. For iron concentration in erythrocytes, an aliquot of erythrocyte samples was mixed with an equal volume of a specific buffer inducing iron release (equal volume of 1.4 M HCl and 4,5% KMnO_4_ in H_2_O). After 2 h incubation at 60 °C and a cooling step at room temperature, 30 µL of iron detection reagent was added (6.5 mM ferrozine, 6.5 mM neocuproine, 2.5 M ammonium acetate and 1 M ascorbic acid dissolved in water). After 30 min of incubation at room temperature, 200 µL of samples was transferred in a well of a 96-well plate and the absorbance was read at 550 nm with a microplate reader (FLUOstar Optima, BMG Labtech, Ortenberg, Germany). The total erythrocyte iron was calculated with respect to the calibration curve established with the ferrous sulfate (FeSO_4_, Sigma-Aldrich, Darmstadt, Germany) (calibration range 25–400 μM) and normalized to hemoglobin content. Results were expressed as µmol of Fe^2+^ per µmol of hemoglobin.

For total iron content determination in HUVECs and EA.hy926, the same protocol was applied on 100 μL of cell lysates. The total intracellular iron was normalized according to total protein content. Results were expressed as µmol of Fe^2+^ per µg of protein.

### 2.12. Heme Assay

Heme content in endothelial cells (HUVECs and EA.hy926) was assessed following the protocol previously described [22]. After stimulation with erythrocyte samples in a 6-well plate, confluent cells were washed three times with PBS and cells were “solubilized” in 1 mL of concentrated formic acid. The heme concentration in the formic acid solution was determined with a spectrophotometer (Secoman Uvline 9600) at 398 nm. Intracellular heme content was calculated with respect to the erythrocyte solution calibration curve (calibration range: 0–2.2 × 10^7^ erythrocytes). The result was expressed as a phagocytic index, corresponding to the quantity of phagocytosed erythrocytes reported to the treated endothelial cell number.

### 2.13. Mitochondrial Oxidative Stress

Mitochondrial oxidative stress was assessed on HUVECs by flow cytometry using Beckman Coulter’s CytoFLEX with MitoSOX Red dye as fluorescent probe, as previously described [23]. Experiments were done in a 12-well plate with each condition performed in triplicate. HUVECs were incubated with 5 mM MitoSOX Red solution after 1 h treatment with modified erythrocytes. Results are expressed as the percentage of labeled cells, relative to the signal obtained for the vehicle control (HUVECs incubated with complete medium).

### 2.14. Permeability Assay

For endothelium permeability determination, HUVECs (3 × 10^5^) were seeded on Millicell^®^ 12-well hanging cell culture inserts (PET membrane with 0.4 μm pores)(Millipore, Burlington, MA, USA). Confluent cells were loaded with isothiocyanate (FITC)-labeled dextran (4 kDa and 70 kDa), prior to and during cell incubation in the absence (control) or the presence of erythrocytes (18.6 × 10^6^ cell/cm^2^). Mannitol hyperosmotic solution (1.4 M) was used as a positive control for endothelial barrier opening. After 6 h of incubation and three successive washes with PBS, 800 μL of 10 kDa or 70 kDa dextran solutions (prepared at 0.385 mg/mL in serum-free RPMI 1640 (without phenol red)) were added on cells (apical chamber). The basolateral chamber was composed of serum-free RPMI 1640 (without dextran at the beginning of the test). Medium from the basolateral chamber was collected and replenished with fresh medium every 15 min for 1 h. The concentrations of FITC-dextran in medium from the basolateral chamber were determined by fluorescence measurements in collected samples with a microplate reader (Fluostar Optima; emission wavelength: 485 nm, excitation wavelength: 520 nm). The concentrations of FITC-dextran in medium from the basolateral chamber were calculated with respect to the FITC-dextran calibration curve (calibration range: 0.001–1 mg/mL). Results were expressed as a permeability increase calculated with the formula P_s_/P_ctrl_ where P_s_ and P_ctrl_ are the slope of the curve corresponding to cumulated FITC-dextran concentration in medium against time for sample and control, respectively.

### 2.15. Immunochemistry Analysis

For fluorescence detection of erythrophagocytosis with pHrodo and PKH67 probes or for immunofluorescence detection of conjugated antibodies, EA.hy926 or HUVECs were seeded on coverslips and incubated for 6 h with or without erythrocytes at a density of 18.6 × 10^6^ cell/cm^2^.

For immunofluorescence, HUVECs were washed three times with PBS and fixed for 20 min in 4% paraformaldehyde solution. Cells were then permeabilized with PBS/0.05% Triton X-100 for 10 min, blocked with PBS/1% BSA for 30 min and were stained with anti-CD31 (Sigma; 1:200 dilution), α-tubulin (Sigma; 1:200 dilution), mouse anti-human VE-cadherin (Santa-Cruz Biotechnology, 1:50 dilution) or mouse anti-human E-selectin (R&D Systems, 1:200 dilution). After 1 h incubation, cells were washed three times with PBS and incubated with secondary antibodies for 2 h. The secondary antibodies used were either a donkey anti-mouse antibody conjugated to Alexa Fluor A488 (Abcam, 1:500), a goat anti-rabbit conjugated to Alexa Fluor 488 (Life technologies; 1:500 dilution) or a rabbit anti-mouse antibody conjugated to Alexa Fluor 488 (Life technologies; 1:500 dilution). Endothelial cells nuclei were stained with Hoechst 33,342 (NucBlue^®^ Live ReadyProbes^®^ Reagent; R37605; Molecular Probes, Thermo Fisher, Madrid, Spain) or with DAPI (Sigma, 0.1 μg/mL) in PBS at room temperature for 20 min. Controls using a non-relevant IgG were included in each set of experiments. Images were acquired using an inverted fluorescence microscope (IX70, Olympus Iberia, L’Hospitalet de Llobregat, Spain), equipped with the CellSens software (Olympus).

### 2.16. Fabrication of a 3D Device Mimicking Microvessels

The microfluidic device used for the in vitro experiments was a Y-shaped channel with an internal circular diameter of 2 mm made by soft lithography of polydimethylsiloxane (PDMS, Sylgard 184; Dow Chemical Co., Midland, MI, USA). A detailed description of the method of fabrication is included in the Supplementary Materials. In brief, the master consists of a rectangular box that features a Y-shaped outward channel with a semicircular profile 2 mm in diameter at its base. The master was printed with a stereolithographic 3D printer (Form 3B printer; Formlabs, Somerville, MA, USA) in Clear V4 resin (Formlabs), because of its high precision and good performance for replication [24]. The master was post-cured by washing it with isopropanol (10 min in the Form Wash tank; Formlabs), dried and cured with UV light (405 nm wavelength) for 30 min at 60 °C. Two masters were needed to conform the two halves of the final device (Supplementary Figure S1).

PDMS was used as the material to replicate the channel thanks to its optical transparency, permeability to gasses, elasticity and biocompatibility [25]. Replication was made by soft lithography of PDMS (curing agent in a ratio of 10:1). PDMS was degassed in a vacuum chamber (40 min at 400 mbar) and cured in an oven (12 h at 60 °C). The two semicircular channels were bonded in a plasma cleaner under oxygen atmosphere (Diener Zepto; Diener, Ebhausen, Germany) and then thermally treated (20 min at 100 °C) to favor full bonding and improve optical quality.

### 2.17. Flow Assay

A functional assay was designed to investigate the possible effects of glycated erythrocytes in human endothelial cells under biomimetical conditions. The flow devices were sterilized following autoclaving at 121 °C for 1 h. After that, the channels of the devices were coated with fibronectin (Gibco) at 5 µg/mL in 0.02% gelatin solution at 37 °C for 3 h. This solution was removed immediately before seeding the cells. Confluent HUVECs were trypsinized (0.25% in Hank’s balanced salt solution with 1 mM EDTA; Gibco, Thermo Fisher Scientific, Waltham, MA, USA) and seeded in the pre-coated PDMS channels at a concentration of 1.5 × 10^6^ cells/mL and maintained in a humidified incubator at 37 °C for 3 h. Then, the devices were flipped over and the seeding process was repeated to coat the cells on the entire channel surface. Once covered with HUVEC, the devices were maintained overnight in the incubator until the start of the fluidic experiment. Perfusion started at 0.5 mL/min with EGM-2 medium to avoid damage to the monolayer, and velocity was doubled every 60 min until reaching 3 mL/min with a peristaltic pump (Ismatec ISM596D Reglo, Ismatec, Wertheim, Germany).

Preparations of erythrocytes, subjected to 5 days glycation with 100 mM D-glucose, were compared to preparations incubated with a physiological concentration of glucose (5 mM). Erythrocytes were introduced in the perfusion circuit under sterile conditions at a concentration of 2.8 × 10^5^ erythrocytes/µL and they circulated at a flow of 3 mL/min for 5 h through the channel device coated with an endothelial cells’ monolayer on the inner wall. Afterwards, the microfluidic chambers were washed with PBS for 5 min to remove any remaining erythrocyte in circulation. The cells were then fixed using paraformaldehyde (PFA) 4% (Ted Pella, INC., Redding, CA, USA) for 10 min and were then washed with PBS. Immunofluorescence staining of protein markers or nucleus staining was then performed. The nuclei of the endothelial cells were stained with Hoechst 33,342 for cell counting on each part of the bifurcation channel and cells were also stained with VE-cadherin and quantified by fluorescence intensity. Three to five fluorescent and contrast phase images (2200 × 1600 µm) from each channel were taken from at least three independent experiments (Olympus IX51, Olympus, Shinjuku, Tokyo, Japan). Images were processed with ImageJ software [26] to calculate the number of cells in the images by counting the number of nuclei.

### 2.18. Numerical Simulation

A detailed description of the numerical methods is included in the Supplementary Materials. In brief, fluid dynamics was analyzed with in silico numerical simulations using Star-CCM+ software [Star-CCM+ documentation] to design geometries, build a grid and carry out the experiments. Fluid dynamics was simulated by the incompressible Navier–Stokes equations described below:

(1) $$∆\vec{v}=0$$

(2) $$\rho (\vec{v}\cdot \nabla )\vec{v}=-\nabla p+\mu ∆\vec{v}$$

where ρ is the fluid density, μ is the fluid viscosity, v ^→^ is the fluid velocity and p is the pressure.

Computations were run until a steady state was reached and convergence monitors were set at 10^−5^. The domain used to perform the numerical simulation was a bifurcation with an opening angle α equal to 90° and sharp vertex (carina). Medium was considered as fluid in experiments and in numerical simulations with a density of 1025 kg/m^3^ and a viscosity equal to 0.0015 Pa·s. Zero pressure was set at the outlets and the inlet boundary condition was defined in order to achieve the same Reynolds number as those reached in the experiments, and thus the same flow behavior. Reynolds number was always less than 100 and thus laminar flow was guaranteed for all the simulations. Rigid erythrocytes were modeled with a biconcave shape with a diameter of 6 µm. The forces acting on erythrocytes were drag and lift force and gravity (see the Supplementary Materials for their mathematical description). An appropriate numeric mesh was established in order to ensure that the results obtained do not depend on the discretization of the geometry (see the Supplementary Materials for a complete description).

### 2.19. Statistical Analysis

All the results are expressed as the mean +/− SEM of multiple experiments. Statistical analysis was achieved using Prism software (Prism software, GraphPad, San Diego, CA, USA). Significant differences (*p* < 0.05) between the means were determined by one-way analysis of variance (ANOVA) procedures followed by Tukey’s multiple comparison test, respectively. In the numerical simulations, the independence of the geometrical mesh employed was assessed using four different types of mesh that showed less than 5% of variation in the magnitude measured (see the Supplementary Materials). This allowed us to consider the differences observed in the results about the time of residence and number of impacts of erythrocytes as statistically significant.

## 3. Results

### 3.1. Glucose-Mediated Glycation Induces Morphological, Structural and Deformability Alterations in Erythrocytes

Erythrocytes were glycated in vitro by incubation at 37 °C for 5 days with 0, 5, 50 or 100 mM of glucose. A previous study performed in our laboratory showed that such in vitro conditions represent a suitable erythrocyte glycation model inducing HbA1c percentages similar to those that can be measured in diabetic patients [16]. In our erythrocyte models, morphologic, alteration and deformability parameters were investigated (the main data are summarized in Table 1).

**Figure 1:**
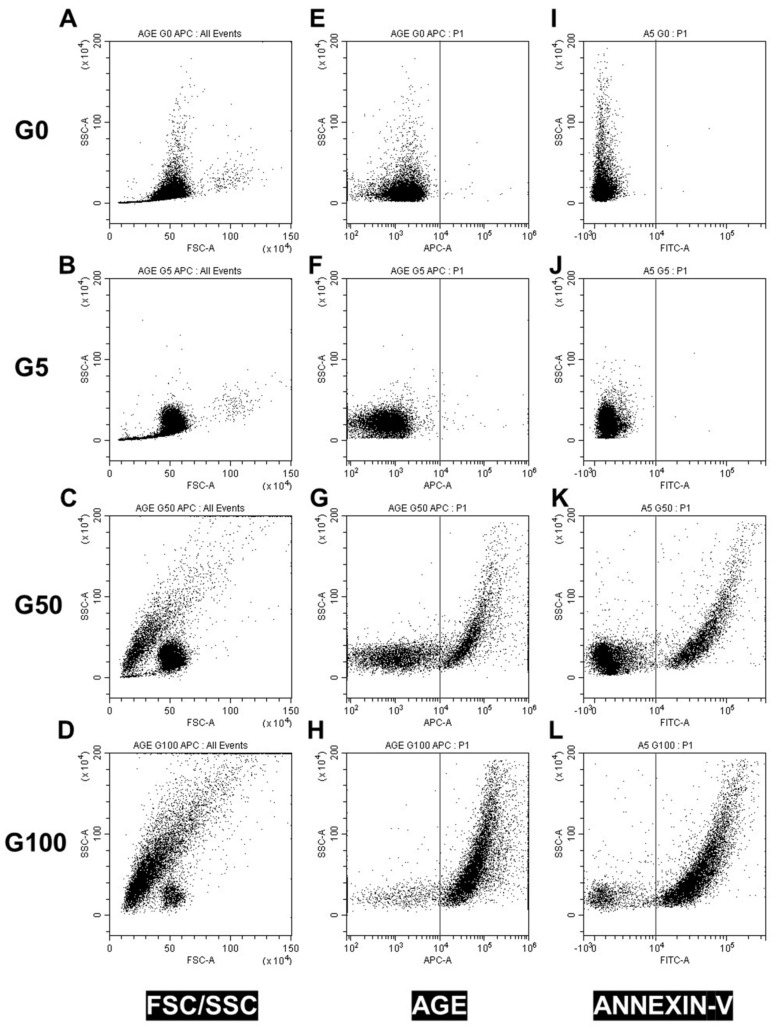
Effect of glucose glycation on erythrocyte morphology, AGE levels and eryptosis. Erythrocyte morphology, AGE level and eryptosis were investigated by flow cytometry. (**A**–**D**) Erythrocyte populations were gated according to cell location in a forward scatter (FSC) versus a side scatter (SSC) parameter. Representative FACS dot plots (FSC/SSC) for (**A**) fresh (G0), (**B**) 5 mM glucose (G5), (**C**) 50 mM glucose (G50) and (**D**) 100 mM glucose (G100) erythrocytes. (**E**–**H**) Advanced glycation end-product levels were assessed by using AGE antibody. Typical representative FACS dot plots after immunostaining of (**E**) G0, (**F**) G5, (**G**) G50 and (**H**) G100 erythrocytes. (**I**–**L**) Phosphatidylserine exposure (PS) was investigated by using annexin V-FITC fluorescent probes. Typical representative FACS dot plots after annexin V staining of (**I**) G0, (**J**) G5, (**K**) G50 and (**L**) G100.

The morphology of erythrocytes was analyzed by FACS by monitoring the FSC and SSC values corresponding to the cell size and granularity, respectively (Figure 1A–D). Glucose-treated erythrocytes at both concentrations showed reduced forward scatter (geo FSC) with and enhanced side scatter (geo SSC), indicating a reduced erythrocyte size associated with a greater morphologic heterogeneity typical of senescent erythrocytes.

Flow cytometry was also used to investigate markers of glycation (AGE) and markers of recognition (PS exposure and CD47) involved in clearance of senescent erythrocytes. A significant proportion of erythrocytes display AGE on their surface when incubated with 50 mM (28.5 ± 8.6% of AGE positive) or 100 mM (72.2% ± 5.3% of AGE positive) of glucose (Table 1/Figure 1E–H). In parallel, these glycated erythrocyte models undergo eryptosis as featured by an increase in phosphatidylserine exposure (PS) for 50 mM glucose-incubated (36.9 ± 5.3% PS positive) and for 100 mM glucose-incubated (76.6 ± 8.4% PS positive) erythrocytes (Table 1/Figure 1I–L).

In addition, glucose treatment with both concentrations showed higher mean fluorescence intensity of CD47, indicating a higher number of erythrocytes displaying the conformational variable epitope (clone 2D6) of CD47 (Table 1/Supplementary Figure S3).

This morphological change in erythrocytes combined with an eryptotic and senescence process cannot be attributed to the sole incubation at 37 °C and to the hypertonic conditions due to glucose concentrations. Indeed, erythrocyte incubation at 37 °C for 5 days with a physiological concentration of glucose (5 mM) did not affect the morphology (Figure 1A,B) and did not induce eryptosis (Figure 1I,J). Moreover, replacing glucose with mannitol during incubation to reach identical hyperosmolar conditions did not induce any alteration of erythrocyte integrity either (Supplementary Figure S4).

Membrane deformability was investigated by ektacytometry according to its resistance to enhanced shear stress intensity. Deformability curves corresponding to elongation index values (EI), as a function of increasing shear stress intensity in isotonic conditions, are featured in Figure S5 (Supplementary Materials). Both pertinent elongation parameters (Ei_max_: EI at infinite shear stress and SS_1/2_: half-maximal deformation) were obtained from these curves (Table 1). Both glucose-treated erythrocyte models exhibit lower Ei_max_ values (0.103 ± 0.019 for G100) and lower SS_1/2_ values (0.104 ± 0.170 for G100) than for non-glycated RBCs (G5) (Ei_max_: 0.243 ± 0.064 and SS_1/2_: 2.742 ± 0.673). Both parameters reflect the loss in the capacity of glycated erythrocytes to be optimally deformed. In parallel with these mechanical alterations, a significant decrease in intracellular iron content was also measured in glycated erythrocytes. Indeed, the number of Fe^2+^ molecules per hemoglobin drops from 3.9 (±0.13) to 2.2 (±0.48) for 100 mM glucose-incubated erythrocytes, corresponding to a 43% decrease (Table 1).

In summary, our glycated erythrocyte preparations exhibit an eryptotic/senescent phenotype rendering them more prone to interact with, be recognized by and be endocytosed by phagocytic cells.

### 3.2. Glycated Erythrocytes Impair Endothelial Cell Homeostasis

**Figure 2:**
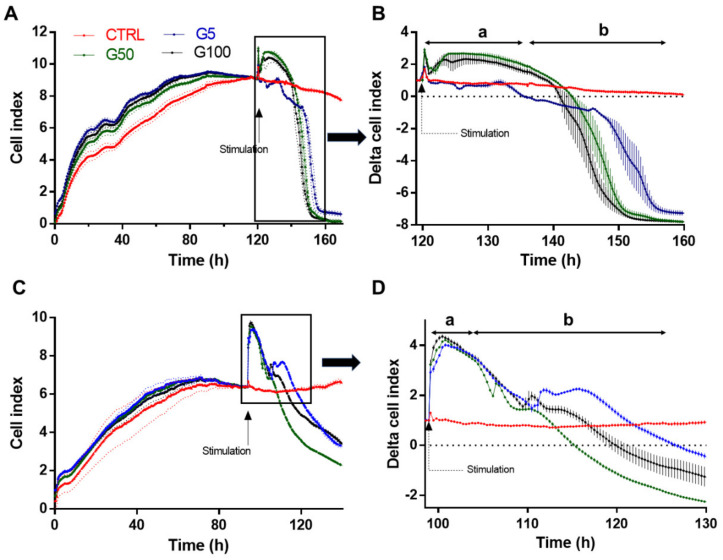
Real-time monitoring of EA.hy926 and HUVECs barrier dysfunction in response to glycated erythrocytes. Immortalized human vascular endothelial cells (EA.hy926, **A**) and human umbilical vein endothelial cells (HUVECs, **C**) (25,000 cells/well) were seeded on gelatin-coated xCELLigence 16 well E-plates. The cell index was recorded continuously until confluence, when a plateau is reached (indicated cell growth in **A**,**C**). Cells were stimulated G5 (blue line), G50 (grey line), G100 (black line), or medium (red line). Cell indexes were normalized after stimulation (as indicated by the arrow in **B**,**D**). Representative results from three independent experiments.

To investigate whether glycated erythrocytes can impact endothelium layer homeostasis, cell real-time electrical impedance was measured in EA.hy926 and HUVECs incubated in the absence (CTRL) or presence of our different erythrocyte preparations (G5, G50 and G100). As shown in Figure 2A,C, a phase of proliferation precedes the plateau, corresponding to the complete endothelial monolayer formation and resulting in a stabilization of the cell index. Then, confluent EA.hy926 cells were stimulated with erythrocytes after 120 h (Figure 2A) and HUVECs after 90 h (Figure 2C).

After this stimulation, two successive phases are observed for cells treated with G5, G50 and G100 erythrocytes (a magnification of the area is shown in Figure 2B,D). There is a first phase of the plateau (a in Figure 2B,D), which lasts about 15 h and 6 h in EA.hy926 and HUVECs, respectively. Cell stimulation by erythrocytes produces a significant increase in the cell index. In EA.hy926 cells, the increase in cell index is particularly evident when cells have been treated with glycated erythrocytes (G50 and G100). Then, during the second phase (phase b), a progressive decrease in the cell index is observed. The drop in cell index is more rapid in both EA.hy926 and HUVECs that have been treated with glycated erythrocytes (about a 10 h shift between G100 and G5).

Altogether, these impedance results clearly show an enhanced interaction and impact of glycated erythrocytes on both endothelial cell models.

### 3.3. Phagocytosis of Glycated Erythrocytes by EA.hy926 and HUVECs

We investigated whether glycated erythrocytes can be rapidly phagocytosed by endothelial cells by using two distinct fluorescent probes allowing erythrocyte tracking: PKH67 and pHrodo. PKH67 is a fluorescent probe that irreversibly binds the erythrocyte membrane. The other probe, pHrodo, is pH-sensitive and integrates the erythrocyte cell compartment and emits fluorescence when present in an acidic environment such as in a phagolysosome (Turpin, 2022 unpublished manuscript).

**Figure 3:**
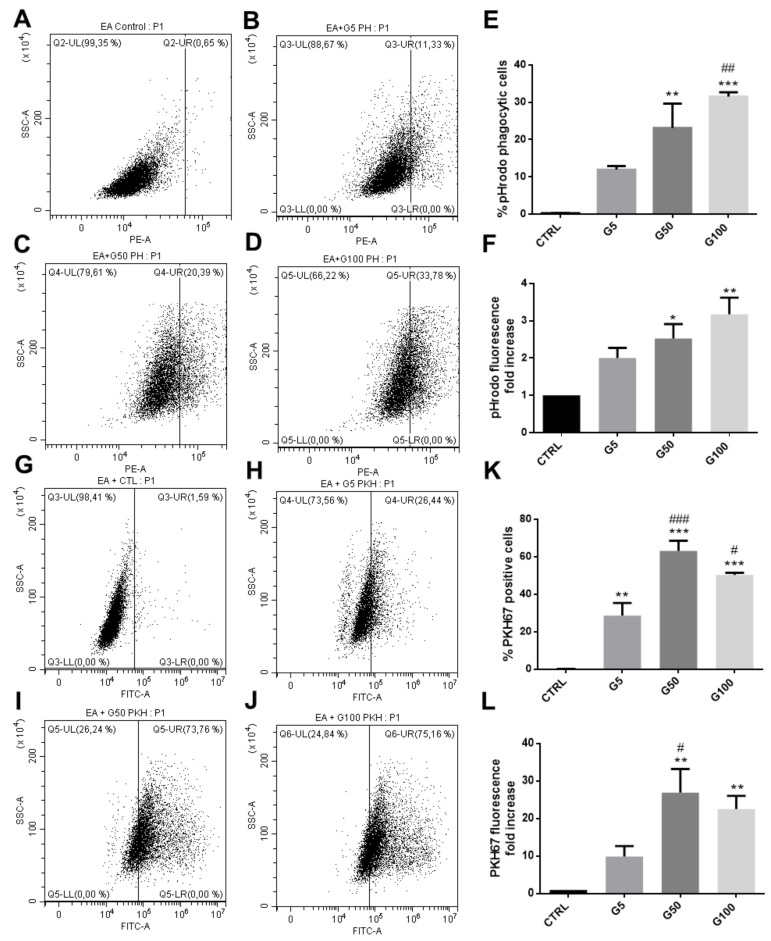
Enhanced erythrophagocytosis of glycated erythrocytes in EA.hy926 cells. Erythrophagocytosis was quantified by FACS using pHrodo and PKH67 fluorescent probes. (**A**–**D**) Representative FACS dot plots after EA.hy926 cell stimulation for 6 h without (**A**) or with pHrodo labeled (**B**) G5, (**C**) G50 and (**D**) G100 erythrocytes. (**E**) Average percentage of pHrodo positive cells (phagocytic cells). (**F**) Average fold increase in pHrodo fluorescence compared with CTRL. (**G**–**J**) Typical representative FACS dot plots after EA.hy926 cell stimulation for 6 h without (**G**) or with PKH67 labeled (**H**) G5, (**I**) G50 and (**J**) G100 erythrocytes. (**K**) Average percentage of PKH67 positive cells (phagocytic cells). (**L**) Average fold increase in PKH67 fluorescence compared with CTRL. Data are the mean ± SEM of five independent experiments. * Effect of GR incubation (vs. CTRL), * *p* < 0.05, ** *p* < 0.01, *** *p* < 0.001; # effect of glycation (vs. G5), # *p* < 0.05, ## *p* < 0.01, ### *p* < 0.001.

After 6 h incubation at 37 °C, FACS was used to investigate the interaction between endothelial cells (EA.hy926) and erythrocytes (glycated or not), previously labeled with pHrodo (Figure 3A–F) or with PKH67 (Figure 3G,L). We showed that about 22% and 30% of EA.hy926 cells exhibit a high fluorescence intensity when previously incubated with pHrodo-labeled G50- and G100-glycated erythrocytes, respectively (Figure 3E). In parallel, the intracellular increase in pHrodo fluorescence suggests that significantly more glycated erythrocytes were phagocytosed by each phagocytic endothelial cell than non-glycated erythrocytes (Figure 3F). Similarly, this enhanced erythrophagocytosis of glycated erythrocytes was also observed by using the PKH67 probe. Indeed, enhanced fluorescence intensity concerns up to 60% of EA.hy926 cells that were incubated with PKH67-labeled glycated erythrocytes (G50) (Figure 3K). Again, the intracellular increase in PKH67 fluorescence suggests that erythrocyte glycation promoted phagocytosis by endothelial cells (Figure 3L).

**Figure 4:**
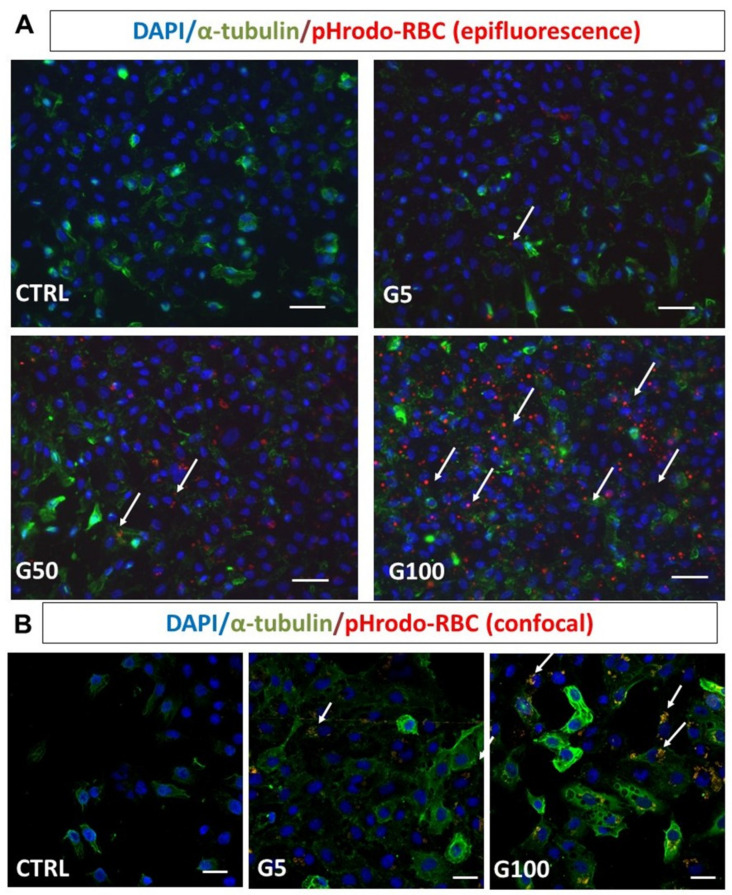
Glycated erythrocytes are phagocytosed by EA.hy926 cells. Human endothelial cells EA.hy926 were incubated without or with pHrodo labeled G5, G50, and G100 erythrocytes for 6 h at 37 °C. After incubation, cells were washed three times with PBS to discard unbound erythrocytes. (**A**) Epifluorescence imaging analysis after immunostaining of EA.hy926 (α-tubulin in green) and erythrocytes (pHrodo in red) after co-incubation without (CTRL) or with G5, G50, or G100 erythrocytes. (**B**) Confocal imaging analysis of EA.hy926 after co-incubation without (CTRL) or with G5 or G100 erythrocytes preliminarily labeled with pHrodo. Cell nuclei were stained with DAPI (blue). White arrows point to phagocytosed erythrocytes, scale bar = 40 μm (**A**) and scale bar = 20 μm (**B**).

The interaction between erythrocytes and EA.hy926 endothelial cells was visualized by epifluorescence microscopy (Figure 4A, pHrodo-labeled erythrocytes in red, DAPI-labeled nucleus in blue). Glycation clearly enhanced erythrocyte interaction with endothelial cells and their subsequent internalization. Confocal microscopy analysis further confirmed the enhanced internalization of glycated erythrocytes by endothelial cells (Figure 4B).

Regarding the interaction of glycated erythrocytes with HUVECs, a preliminary experiment was performed directly on umbilical cord explants. One of the two umbilical arteries was filled with G50 glycated erythrocytes and incubated for 3 days. The presence of erythrocytes and their interaction with the artery endothelium were analyzed in the human cord section (Figure S6, Supplementary Materials). After intensive washing of the artery, we observed that many erythrocytes remained bound to the vessel wall, as shown by the white arrowheads (Figure S6C, Supplementary Materials). The presence of the erythrocytes in the close vicinity of the endothelium indicates their high interaction with endothelial cells and their putative (erythro)phagocytosis.

**Figure 5:**
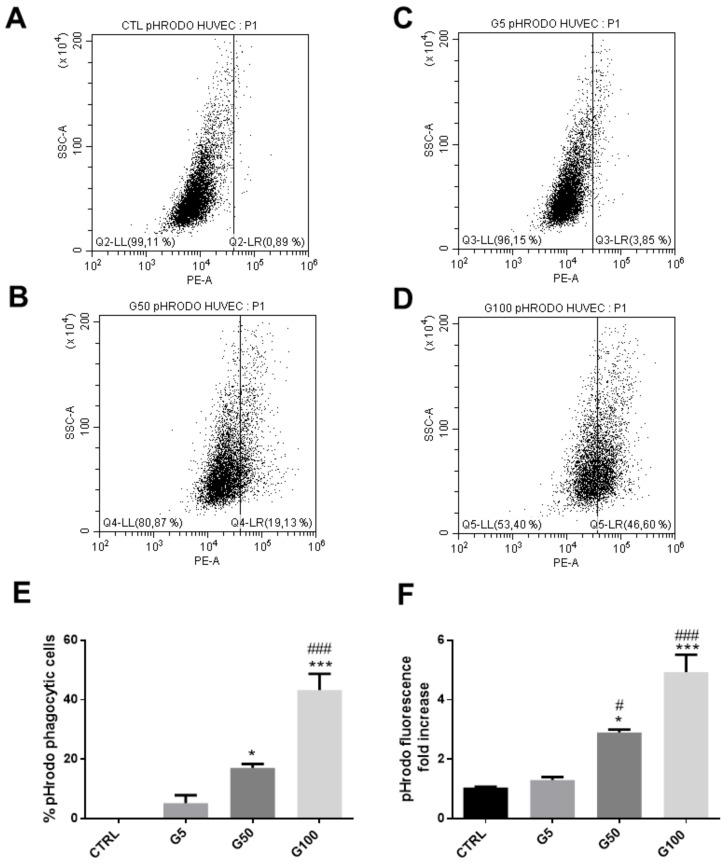
Enhanced erythrophagocytosis in HUVECs when incubated with glycated erythrocytes. Erythrophagocytosis was evaluated by FACS using pHrodo fluorescent probe. (**A**–**D**) Representative FACS dot plots after HUVEC stimulation for 6 h without (**A**) or with pHrodo labeled (**B**) G5, (**C**) G50, and (**D**) G100 erythrocytes. (**E**) Average percentage of pHrodo positive cells (phagocytic cells). (**F**) Average fold increase in pHrodo fluorescence compared with CTRL. Data are the mean ± SEM of five independent experiments. * Effect of erythrocyte incubation (vs. CTRL), * *p* < 0.05, *** *p* < 0.001; # effect of glycation (vs. G5), # *p* < 0.05, ### *p* < 0.001.

In addition, we showed that HUVECs also exhibited an enhanced phagocytic activity for glycated erythrocytes. As illustrated in Figure 5, more than 40% of HUVECs exhibited an erythrophagocytosis when incubated with pHrodo G100-glycated erythrocytes (Figure 5E). The presence of pHrodo-labeled glycated erythrocytes within HUVECs after 6 h incubation can be clearly visualized by epifluorescence microscopy (Figure S7, Supplementary Materials). These results confirm the enhanced propensity of glycated erythrocytes to be phagocytized by endothelial cells. It is important to note that phagocytosis of glycated erythrocytes does not occur in all cell models. For example, Vero cells did not exhibit any erythrophagocytosis capacity as shown by the absence of pHrodo positive cell population when treated with glycated erythrocytes (Figure S8, Supplementary Materials).

### 3.4. Impact of Erythrophagocytosis on Endothelium Permeability

In our experimental conditions, the increased erythrophagocytosis observed in EA.hy926 and HUVECs when incubated with glycated erythrocytes may have a deleterious impact on their main intrinsic functions, such as endothelial barrier function. HUVEC viability was first investigated by flow cytometry (Figure S9, Supplementary Materials). Six hours of incubation with glycated erythrocytes did not affect cell viability as no significant increase in the percentage of annexin V and propidium iodide positive endothelial cells was noted. Then, the barrier permeability of the HUVEC monolayer was investigated by using an intermediate (10 kDa) and a large-size (70 kDa) fluorescent tracer (FITC-dextran, FD10 and FD70, respectively).

**Figure 6:**
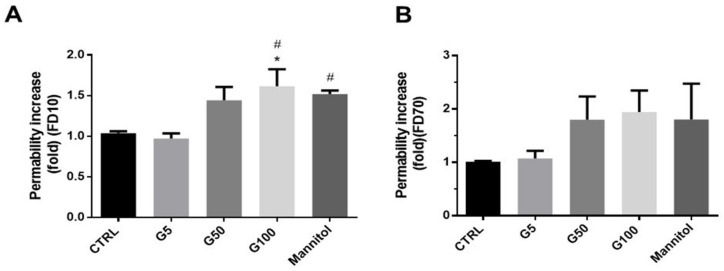
Glycated erythrocytes increase endothelial barrier permeability in HUVECs. Permeability was assayed on HUVECs using (**A**) 10 kDa FITC-dextran (FD10) and (**B**) 70 kDa FITC-dextran (FD70) before and after 6 h treatments with G5, G50, or G100 erythrocytes. Mannitol diluted in DMEM (1.4 M) was used as positive control. The intensity of FITC fluorescence was measured in each basolateral chamber every 15 min after the addition of FD10 or FD70 in the apical chamber. Bars represent the mean ± SEM of the fold increase in permeability (vs. CTRL) (n = 4–5). * Effect of erythrocyte (vs. CTRL), * *p* < 0.05; # effect of erythrocyte glycation (vs. G5), # *p* < 0.05.

By using the intermediate tracer (FD10), a significant increase in the HUVEC permeability was observed when treated for 6 h with G100-glycated erythrocytes (1.5-fold vs. control) (Figure 6A). When using the large-size tracer, the increase in the HUVEC permeability of G100-treated cells did not reach statistical significance (Figure 6B). These results suggest that glycated erythrocytes increase vascular permeability by inducing the formation of small gaps in the endothelium, allowing the passage of small proteins. This gap formation does not appear to be mediated by the adherens junction protein VE-cadherin, whose expression was not altered in our experimental conditions (Figure S10, Supplementary Materials). Similarly, selectin, an important endothelial cell adhesion molecule, was not modified in HUVECs stimulated with glycated erythrocytes (Figure S11, Supplementary Materials).

### 3.5. Erythrophagocytosis Consequences on Iron Accumulation and Oxidative Stress in Endothelial Cells

**Figure 7:**
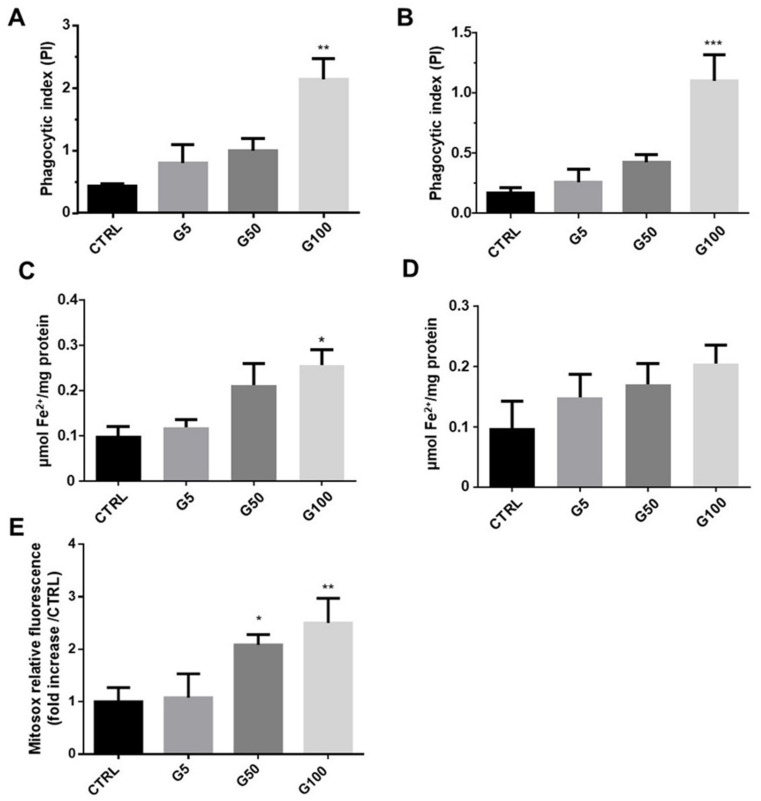
Erythrocyte phagocytosis is associated with an accumulation of intracellular Fe^2+^, heme, and oxidative stress (OS) in HUVECs and EA.hy926 cells. (**A**,**B**) Phagocytic index was determined from the heme colorimetric assay in EA.hy926 and HUVECs, respectively. (**C**,**D**) Intracellular iron concentration was determined by ferrozine colorimetric assays in EA.hy926 and HUVEC cells, respectively. (**E**) Mitochondrial ROS level was expressed as MitoSOX relative fluorescence in HUVECs. Data are the mean ± SEM of five independent experiments. * Effect of erythrocyte incubation (vs. CTRL), * *p* < 0.05, ** *p* < 0.01, *** *p* < 0.001.

Heme and intracellular iron content were measured in endothelial cells (EA.hy926 and HUVECs) incubated in the absence or presence of our different preparations of erythrocytes. As shown in Figure 7A,B, a significant increase in phagocytic index (at least two-fold increase compared to the G5 condition) was observed in endothelial cells incubated with G100-glycated erythrocytes, attesting a significant accumulation of intracellular heme after only 6 h in EA.hy926 and HUVECs. The increased intracellular heme content upon glycated erythrocyte treatment is associated with a marked and significant intracellular Fe^2+^ accumulation in EA.hy926 cells (Figure 7C). In HUVECs, this increase did not reach statistical significance (Figure 7D). The ferrous ion (Fe^2+^) can participate in oxidative stress (OS) production by catalyzing ROS formation. In light of this, oxidative stress induced by incubation of HUVECs with erythrocytes was investigated by flow cytometry by using a MitoSOX probe (Figure 7E and Figure S12, Supplementary Materials). Significant enhancement in mitochondrial superoxide anion production was observed in HUVECs treated with G50- and G100-glycated erythrocytes for a short-term exposure (1 h). These last results highlight the impact of increased erythrophagocytosis on the redox homeostasis of endothelial cells.

### 3.6. Impact of Glycated Erythrocytes on Endothelial Cells under Flow Conditions

**Figure 8:**
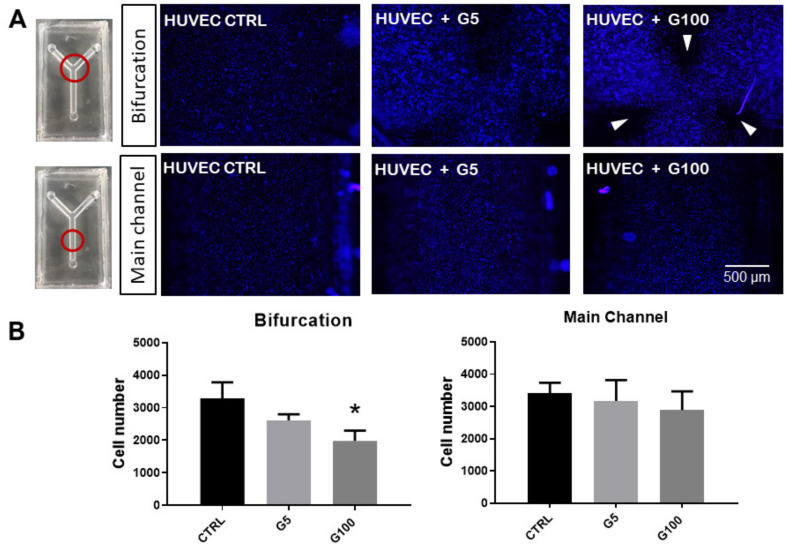
Impact of glycated erythrocytes on HUVECs in flow conditions. (**A**) Representative images of HUVECs covering the inner walls of the fluidic devices, stained with Hoechst 33,342 (nuclei) after flow assays where no erythrocytes (CTRL), normally glycated erythrocytes (G5), and highly glycated erythrocytes (G100) were circulated in the channels for 5 h at 3 mL/min. Images are taken at the bifurcation or in the main channel of the fluidic device as indicated by the red circles in the image. White arrows indicate regions lacking HUVECs at the end of the experiments. (**B**) Quantification of the number of cells for each experimental condition at both parts of the device (bifurcation and main channels). Columns represent the mean value ± SEM (in vertical bars) of at least three independent experiments. * *p* < 0.05 with respect to control.

A functional assay was performed to investigate the possible effects of glycated erythrocytes on human endothelial cells under flow conditions. As shown in Figure 8, the endothelial cell monolayer was normal and “stable” during the control experiments (no erythrocytes circulating in the channel device), both in the main channel and at the bifurcations.

When G5-glycated erythrocytes were introduced in the flowing medium, a non-significant decrease in endothelial cells present at the bifurcation and main channel levels was noted. Interestingly, when G100-glycated erythrocytes were incorporated into the flow, a significant decrease in HUVEC number was measured at the bifurcation of the channels, and more specifically at three specific points of the bifurcation (white arrows in Figure 8): at both outer sides of the bifurcation and in front of the vertex. These results suggest that only glycated erythrocytes affect HUVEC integrity, and only at these specific points of the channel bifurcation. It is important to note that impact of glycated erythrocytes on HUVEC monolayer integrity was not associated with an adherens junction barrier dysfunction as shown by the absence of any statistically significant changes in VE-cadherin expression at any part of the channel device following circulation of glycated or non-glycated erythrocytes (Figure S13, Supplementary Materials).

In order to analyze the possible role of the physical parameters derived from the flow characteristics in the interaction between circulating erythrocytes and endothelial cells in the walls, an in silico numerical simulation was performed. For these “in silico” experiments, a numerical simulation was carried out with an opening angle α = 90°. The geometry was scaled to fit our experiments (with radius R = 8 × 10^−5^ m) and to test high concentrations of erythrocytes, thus optimizing the available computational resources.

**Figure 9:**
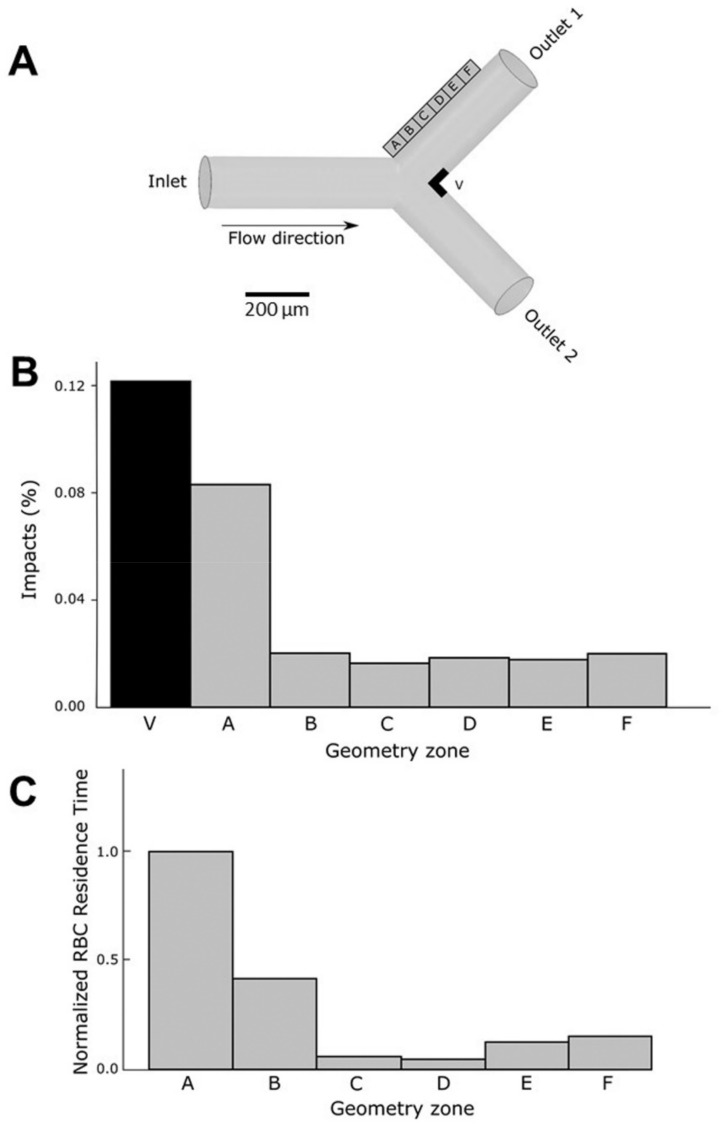
In silico analysis of the impacts of erythrocytes on the walls of the channel under flow conditions. (**A**) Description of the geometry and the regions of analysis. Regions in the outer wall of the bifurcation (A to F) and close to the vertex (V) are defined in order to study erythrocyte movement and interaction with endothelial cells in the walls. (**B**) The number of impacts of the erythrocytes (as a percentage) on the wall was measured in all the regions of the outer wall (A to F) and in the vertex (V) of the bifurcation. (**C**) Erythrocytes (RBC) residence time in regions A to F of the outer wall expressed as the normalized ratio with respect to residence time in region A (the region with highest residence time). Residence time of erythrocytes in region V is almost null.

As is shown in Figure 9A, six different regions (labeled A to F) were studied at the outer wall of the daughter vessel (the other branch exhibited a symmetrical behavior) and the impacts and residence time were defined as relevant variables to determine the interaction of the erythrocytes with the endothelial cells at the wall level. At the same time, a region was defined close to the carina of the bifurcation as this area was shown to be affected by inertial trajectories of the erythrocytes [27]. Supplementary Videos S1 and S2 (Supplementary Materials) show the behavior of the erythrocytes in the flow.

Figure 9B shows a plot of the percentage of erythrocytes that reach each area region close to the outer wall and the vertex (carina). As expected, the largest percentage of erythrocytes that touch the wall was obtained in the area of the vertex (V). More interesting were the results concerning the outer wall. In region A, the closest to the bifurcation, a larger number of impacts were monitored, whereas in the other regions (B to F) erythrocyte impacts were significantly lower.

The residence time of erythrocytes in a specific region constitutes a determinant parameter since the longer the interaction time of the erythrocytes with the walls, the higher the detrimental incidence on the endothelial cells at the arterial wall. Figure 9C represents the residence time at different locations of the outer wall. The residence time at the carina region (V) was not represented because of the very low residence time due to the large velocities reached at this specific place. The longest residence time was determined in section A, corresponding to the area of the bifurcation. As seen in video simulation (Supplementary Video S2), bifurcations are characterized by the presence of low velocity zones at the outer wall, allowing longer periods of interaction between circulating erythrocytes and endothelial cells.

These in silico results of the impact of erythrocytes on endothelial cells under flow conditions can easily give explanatory elements of the experiments performed in vitro (Figure 8). Increased residence time of erythrocytes at bifurcations would induce a longer interaction with endothelial cells. Moreover, erythrocytes when glycated are more prone to interact with endothelial cells, be phagocytosed and alter the integrity at these endothelial sensitive regions.

## 4. Discussion

In this study, a glycated erythrocyte model was established following pathological conditions that can be encountered in diabetic patients. Incubation with high concentrations of glucose (until 100 mM) at 37 °C for a period of 5 days was chosen to reproduce conditions occurring during blood stasis in the neovessels of atherothrombotic plaques. Recent publications from our group revealed that such in vitro conditions induced HbA1c percentages and cell characteristics quite similar to those that can be measured in diabetic patients [15,16].

Here, we confirm that erythrocyte morphology following glycation was characterized by a reduced size and heterogeneous shapes associated with a drastic loss of deformability. Among the key cytoskeleton proteins involved in the membrane flexibility, β-actin was shown to be glycated upon exposure to high glucose concentrations, and associated with a decrease in erythrocyte deformability [28]. Other important membrane proteins, such as ankyrin, band 3 or glycophorin, are closely linked to erythrocyte deformability and are also prone to glycation [29]. As indicated by the reduced FSC value and enhanced SSC value, glycation causes cellular shrinkage in erythrocytes and membrane blebbing, which correspond to peripheral cell irregularities. This explains the heterogeneity in the morphology of erythrocytes [30]. Cell shrinkage and membrane blebbing constitute typical hallmarks of senescent or eryptotic erythrocytes [31].

As for senescent cells, we showed that glycated erythrocytes expose “eat-me” signals on their surface for their recognition and subsequent elimination by phagocytes. In particular, a high AGE expression was featured on erythrocytes when glycated. AGEs were shown to promote erythrocyte interaction with many cells such as endothelial cells, expressing their receptors such as RAGE or CD36 [32]. In parallel, we showed that glycation triggers eryptosis as evidenced by the enhanced phosphatidylserine exposure at the erythrocyte surface. Glucose-mediated glycation can impair erythrocyte pump activity, and specifically the Ca^2+^-ATPase pump allowing the active transport of Ca^2+^ across the erythrocyte cell membrane. The activation of this pump upon glycation induces a massive intracellular calcium accumulation causing cell shrinkage and phosphatidylserine exposure [33,34]. Interactions between glycated erythrocytes and endothelial cells can occur through phosphatidylserine recognition as reported in numerous studies [35,36]. CXCL16 represents the main scavenger receptor for phosphatidylserines involved in eryptotic erythrocyte clearance by endothelial cells, but many other phosphatidylserine receptors have been identified [37]. In addition, following erythrocyte glycation, the CD47 senescence marker has been shown to undergo a conformational change as indicated by the increased expression in CD47 clone 2D6 reflecting a variable domain of the protein. This conformational change in CD47 promotes the specific recognition for glycated erythrocytes as an “eat me” signal by potential phagocytic cells [38]. Indeed, the conformational change of CD47 allows its binding to thrombospondin-1 (TSP-1) and the subsequent recognition of the complex formed (CD47-TSP1) by the signal regulatory protein α (SIRPα), regulating phagocytosis [38].

The differential expressions of surface markers such AGEs, PS and CD47 in erythrocytes following glycation can clearly trigger their recognition by endothelial cells. Here, we showed that interaction between glycated erythrocytes and endothelial cells occurs rapidly and impacts cell homeostasis as indicated by cell impedance results performed on both EA.hy926 and HUVECs. Indeed, cell stimulation by erythrocytes produces a significant increase in the cell index in the first 6 h, suggesting an interaction of glycated erythrocytes with the endothelial cells. This interaction cannot be attributed to a simple adhesion of erythrocytes with endothelium, but rather to an enhanced erythrophagocytosis phenomenon in endothelial cells observed after only 6 h of exposition with glycated erythrocytes. Although in direct contact with circulating blood compounds, endothelial cells were not considered as specialized phagocytic cells involved in senescent erythrocyte clearance. However, like other cell types such as smooth muscle cells (SMCs), endothelial cells can bind and ingest aged or senescent erythrocytes [15,32]. In addition, and by using DAF as a colorimetric method of detection of internalized erythrocytes, recent studies from our group reported endothelial cell (EA.hy926) capacity to preferentially phagocytose glycated erythrocytes after 24 h incubation [15,16]. In the present work, we highlighted the internalization of erythrocytes using fluorescence probes (pHrodo and PKH67), which allow a more quantitative and specific detection of erythrophagocytosis than DAF reagent.

In relation to the different markers identified on glycated erythrocytes, many pathways may be involved in the phagocytic mechanism. Erythrocyte–endothelium interaction can be mediated via the AGE–RAGE axis as shown by Wautier et al. on HUVEC primary cultures [32]. The recognition of phosphatidylserine from eryptotic erythrocytes by their specific cell receptors may be a parallel mechanism for the erythrophagocytic process. Finally, the CD47-TSP1-SIRPa axis constitutes a possible pathway requiring in depth investigations.

In our experimental conditions, increased and early erythrophagocytosis of glycated erythrocytes led to deleterious impacts on the endothelial layer integrity. Indeed, upon glycated erythrocyte incubation, the endothelium became permeable to proteins of 10 kDa or below. VE-cadherin is a component of endothelial cell-to-cell adherens junctions that are associated with the impermeability along with tight and gap junction proteins [39]. Though VE-cadherin is known to be involved in the endothelial barrier functioning, its expression was not altered in our experimental conditions. Other junction proteins such as ZO-1 or occludin for tight junctions or connexins for gap junctions could be investigated. This endothelial activation and dysfunction, observed through the prism of altered permeability, could also induce the expression and secretion of adhesion molecules such as VCAM-1, ICAM-1 and E-selectin [40]. To extend this study, it would therefore be relevant to determine whether endothelial activation occurs with erythrophagocytosis and exacerbates erythrocyte adhesion at the endothelial surface.

Iron is known to play a key role in the atherosclerosis process, particularly in the activation of the endothelium [41,42]. Erythrocyte hemoglobin contains most of the iron in the body (70%), which allows erythrocyte the capacity to carry oxygen. The four iron atoms present in each hemoglobin molecule can bind four oxygen molecules. In our experimental conditions, we showed a drastic decrease in iron content together with a marked reduction in the iron/hemoglobin molar ratio in glycated erythrocytes. The overall loss of iron can easily be explained by the increased hemolysis due to erythrocyte fragility following glycation. By contrast, the decrease of the iron/hemoglobin molar ratio in glycated erythrocytes described here for the first time is quite unexpected. To explain this result, we could hypothesize that glycation impacts the attachment of heme groups to globin units. Indeed, we observed that the heme/hemoglobin molar ratio was reduced following erythrocyte glycation (data not shown). It is worth noting that the lack of heme iron in glycated erythrocytes could lead to deleterious functional consequences.

Despite iron deficiency in glycated erythrocytes, an enhanced intracellular accumulation of the metal was measured in endothelial cells following erythrophagocytosis. Indeed, we showed an intracellular increase in heme associated with iron accumulation in EA.hy926 and HUVECs incubated with glycated erythrocytes. It is worth noting that the measured increase in intracellular iron content was consistent with the heme content of endothelial treated cells. Indeed, this accumulation of iron results from heme degradation by heme oxygenase following erythrophagocytosis [43,44]. Then, the iron released from the heme molecule may not be taken up by ferroportin to be exported extracellularly [45]. In glycated erythrocyte-treated endothelial cells, these high levels of cellular iron probably participate in the overproduction of ROS via the Fenton reaction. Here, we report an overproduction of mitochondrial superoxide radical anion, the precursor of many other ROS. As described in numerous studies, this production of superoxide radical anion and other related ROS constitute relevant contributors to the endothelial dysfunction we observed [46,47]. Indeed, ROS can react with NO produced by endothelial cells, forming the highly oxidant species ONOO. NO bioavailability then becomes dramatically reduced, causing platelet aggregation and enhanced vasoconstriction [48]. Enhanced oxidative stress was also shown to induce expression of adhesion molecules by endothelial cells promoting immune cell recruiting and inflammation [49].

As far as the impact of glycated erythrocytes on endothelial cells is concerned, one should note that cells did not undergo any apoptotic or necrotic processes. We suppose that a prolonged exposure to glycated erythrocytes could impact the migration and proliferation capacity of endothelial cells as previously shown by our group [15]. Cytotoxic effects of glycated erythrocytes occurred following 24 h incubation with EA.hy926 and more rapidly in HUVEC treated cells. Here, a rapid increased interaction between erythrocytes and endothelial cells and subsequent deleterious erythrophagocytosis phenomenon were observed following glycated erythrocyte incubations.

The cell impedance results highlight an early apoptotic process appearing in the first 24 h of incubation with glycated erythrocytes. When glycated erythrocytes rapidly induced endothelial cell apoptosis, cell mortality was detected after 24 h incubation. In our static model, the cytotoxicity observed may be due to the progressive sedimentation of erythrocytes on the endothelial cell layer, impairing an optimal access to oxygen. This prolonged contact between erythrocytes and endothelial cells mimics conditions that can be encountered in the intraplaque neovessel where blood flow is minimal [15].

Still in an attempt to mimic conditions that can be encountered in vivo, effects of native or glycated erythrocytes on endothelial cells were tested under flow dynamic conditions. Therefore, we designed a flow assay to test the effects of our erythrocyte preparations on human endothelial cells covering the inner surface of a biomimetic channel, under flow conditions [50]. To demonstrate the possible influence of hemodynamics, a channel mimicking a Y-shape bifurcation was designed. Compared to the main straight channel, bifurcations promote higher shear stress changes that can impact endothelial cell response [51,52]. In these experimental conditions, glycated, but not native, erythrocytes induced cell detachment from the inner surface of the channel after 5 h of flow exposure. Interestingly, endothelial cell detachment was localized at specific points of the bifurcation, but not in the main straight segment of the channel. The outer sides and the carina of the bifurcation are points of significant wall shear stress, making them particularly susceptible to circulating compounds [51,53]. To quantify the effects of circulating erythrocytes on the endothelial cells at the walls of the channel, in terms of number of impacts and time of residence, a numerical simulation approach was implemented. This simulation in silico reproduced the conditions of our in vitro flow assay. The results were consistent with fluid behavior at the bifurcations. These types of geometries are characterized by the presence of low-velocity zones at the outer wall [51]. These low velocity values allow erythrocytes to interact for longer periods with the endothelial cells at the wall surfaces. At the carina level, the calculated number of impacts of erythrocytes was rather higher than in the rest of the bifurcation. The long exposure together with the high impact number of glycated erythrocytes correlate with the endothelial damage extent observed at the specific points of the bifurcation. For the first time, our results highlighted here a deleterious impact of glycated erythrocytes at specific zones of artificial microvessels, the bifurcation, rendering them more prone to the occurrence of vascular complications.

## 5. Conclusions

The study presented here reveals several novel insights with respect to the impact of glycation on erythrocyte structure and capacity to interact with endothelial cells. Enhanced phagocytosis of glycated erythrocytes may impact endothelial cell function with putative consequences on the development of diabetic vascular complications. Further experiments would be needed to reach better mechanistic insights on the impact of glycated erythrocytes on endothelial cell dysfunctions and to contribute to the research of therapeutic strategies against vascular disorders in diabetes.

## Figures and Tables

**Table 1:**
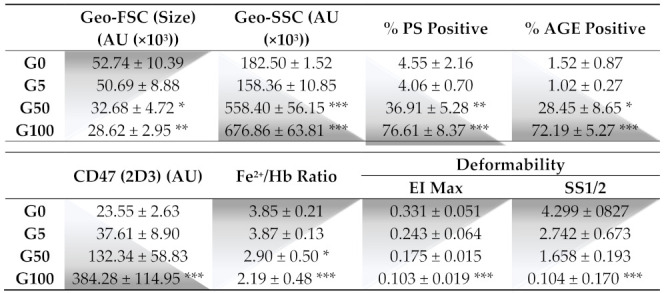
Effect of glucose glycation on morphological, structural and deformability parameters of erythrocytes.

Morphology parameters (Size (forward scatter, FSC), granulosity (side scatter, SSC)), phosphatidylserine exposure (PS), AGE and CD47 levels were determined by flow cytometry. Fe^2+^/Hb molar ratio was determined by ferrozine (Fe^2+^) and Drabkin’s (Hemoglobin) colorimetric assays. Deformability parameters: maximum elongation index (Eimax) and shear stress values applied at the half elongation (SS_1/2_) were calculated from the deformability curves obtained by ektacytometry. Data are the mean ± SEM of 3–5 independent experiments. * Effect of glycation (vs. G5), * *p* < 0.05, ** *p* < 0.01, *** *p* < 0.001.

## Data Availability

Not applicable.

## Supplementary Materials

The following supporting information can be downloaded at: https://www.mdpi.com/2073-4409/11/14/2200/s1. Table S1: Specifications of each grid for the geometry, Figure S1: Stereolithography printer working principle, Figure S2: Red blood cell formed by 54 spheres, Figure S3: Glucose-induced conformational change of CD47 in erythrocytes, Figure S4: Mannitol-induced hyperosmolarity did not affect erythrocyte integrity, Figure S5: Glucose-induced impairment of erythrocytes deformability, Figure S6: Glycophorin A/CD31 and von Willebrand factor (vWF) immunostaining in human artery sections from umbilical cord incubated with glycated erythrocytes (G50), Figure S7: Erythrocyte glycation by glucose-induced erythrophagocytosis in HUVEC cells, Figure S8: Erythrocyte glycation by glucose did not induce erythrophagocytosis in VERO cells, Figure S9: Erythrophagocytosis did not impact HUVEC cells viability, Figure S10: Erythrophagocytosis did not modulate VE-cadherin expression in HUVEC cells, Figure S11: Erythrophagocytosis did not modulate E-selectin expression in HUVEC cells, Figure S12: Induction of mitochondrial oxidative stress by G50 and G100 erythrocytes, Figure S13: VE-Cadherin expression in HUVEC after flow experiments with glycated or non-glycated erythrocytes, Video S1. The movement of the erythrocytes by the simulation, Video S2: Zoom views of the bifurcation and of the main straight portions of the channels. References [54,55,56,57] are cited in the supplementary materials.
