# Supplementary material for: Impact of Enhanced Phagocytosis of Glycated Erythrocytes on Human Endothelial Cell Functions
Source: Cells. 2022 Jul 14;11(14):2200. doi: 10.3390/cells11142200 (PMC9351689; doi:10.3390/cells11142200)
Supplement: Supplementary file 1 [file cells-11-02200-s001.zip › cells-1760787-supplementary.pdf]

Supplementary Materials

Impact of Enhanced Phagocytosis of Glycated Erythrocytes on Human Endothelial Cell Functions

Chloé Turpin [1], Marie Laurine Apalama [1], Bastian Carnero [2,3], Alberto Otero-Cacho [4,5], Alberto P. Munuzuri [4], Maria Teresa Flores-Arias [2], Erick Vélia [6], Olivier Meilhac [1,7], Emmanuel Bourdon [1,*], Ezequiel Álvarez [8,9,10] and Philippe Rondeau [1,*]

1 UMR 1188 Diabète Athérothombose Thérapies Réunion Océan Indien (DéTROI), INSERM, Université de La Réunion, 97400 Saint-Denis, France; chloe.turpin@univ-reunion.fr (C.T.); marie.apalama@gmail.com (M.L.A.); olivier.meilhac@inserm.fr (O.M.)
2 Photonics4Life Research Group, Applied Physics Department, Faculty of Physics, Institute of Materials (iMATUS), Universidade de Santiago de Compostela, 15782 Santiago de Compostela, Spain; bastian.carnero.groba@usc.es (B.C.); flores@usc.es (M.T.F.-A.)
3 BFlow S.L., Edificio Emprendia, 15782 Santiago de Compostela, Spain
4 Galician Center for Mathematical Research and Technology (CITMAga) and Group of Nonlinear Physics, Department of Physics, Universidade de Santiago de Compostela, 15782 Santiago de Compostela, Spain; alberto.otero.cacho@usc.es (A.O.-C.); alberto.perez.munuzuri@usc.es (A.P.M.)
5 FlowReserve Labs S.L., Edificio Emprendia, 15706 Santiago de Compostela, Spain
6 Clinique Sainte-Clotilde, Groupe Clinifutur, Pôle Mère Enfant, 97490 Sainte-Clotilde, France; erick.velia@clinifutur.net
7 Centre Hospitalier Universitaire de La Réunion, 97400 Saint Denis, France
8 Cardiology Group, Health Research Institute of Santiago de Compostela (IDIS), Hospital Universitario de Santiago de Compostela (SERGAS), Trav. Choupana s/n, 15706 Santiago de Compostela, Spain; ezequiel.alvarez.castro@gmail.com
9 Centro de Investigación Biomedica en Red de Enfermedades Cardiovasculares (CIBERCV), 28029 Madrid, Spain
10 Departamento de Farmacología, Farmacia y Tecnología Farmacéutica, Universidade de Santiago de Compostela, 15782 Santiago de Compostela, Spain
* Correspondence: emmanuel.bourdon@univ-reunion.fr (E.B.); rophil@univ-reunion.fr (P.R.)

Supplementary Materials

Material and methods

Fabrication of a 3D in vitro artery-mimicking circular channel device

The master of the device was printed by stereolithography (SLA) with a 3D printer. SLA printers have a tank where a liquid printing resin is contained so a laser source can perform a selective layer-by-layer photopolymerization (Supplementary Figure S1a). In particular, a Form 3B printer (Formlabs, Somerville, Massachusetts) was used. This SLA printer employs Low Force Stereolithography (LFS), a novel technique that improves the precision and accuracy of printed pieces, by reducing the manufacturing stresses that the pieces suffer during the printing. Clear V4 resin was selected as printing resin for fabricating the master given the high precision it offers (a step in Z of 25 μm) and its good performance when replicating polymers [1]. The post cure of the master consists of two steps. First, it is washed in isopropanol (IPA) >90% inside the Form Wash tank (Formlabs, Somerville, Massachusetts) for 10 minutes. Secondly, the piece is left to dry and introduced into the UV Form Cure chamber (Formlabs, Somerville, Massachusetts) for 30 minutes at 60°C, provided with LEDs emitting at a wavelength of 405 nm. The master, designed as a 50x33x10mm rectangular box (with walls added to avoid losses of liquid polymer), features an Y-shaped outward channel with semicircular profile of 2 mm of diameter at its base (Supplementary Figure S1b). Three cylindrical connectors (outward or inward in each case) were added to grant the proper coupling of the two halves of the channel that will compound the final device.

The polydimethylsiloxane (PDMS) polymer was selected as material for performing the final device due to its optical transparency, permeability to gases, elasticity, and biocompatibility [2]. The technology for replicating the master was the soft lithography of polydimethylsiloxane (PDMS). The PDMS used was the Sylgard 184 elastomer (Dow Chemical Company, Midland, Michigan), that is obtained by mixing the monomer with the curing agent in a ratio 10:1. Once the master is printed (Supplementary Figure S1c), the mixture is deposited inside, forming a sheet of 4 mm and introduced in a vacuum chamber 40 min at 400 mbar to remove bubbles produced during the mixing process. Finally, the degassed PDMS inside the master was cured in an oven for 12h at 60°C and peeled off (Supplementary Figure S1d), obtaining a Y-shaped inward channel replica in each iteration.

The final Y-shaped (Supplementary Figure S1e) fluidic device with the internal circular channel is obtained by bonding two matching replicas of the semicircular channel using a Diener Zepto plasma cleaner (Diener, Ebhausen, Germany under oxygen atmosphere). Oxygen plasma procedure transforms the hydrophobic surface of the PDMS, with a surface energy around 20-30 mJ m^{-2}, into a biocompatible hydrophilic surface of around 60-80 mJ m^{-2}, that also improves cell adhesion [3]. The complete Y-shaped device was introduced into a furnace to receive a thermal treatment of 20 minutes at 100 ºC to promote full bonding and improve its optical quality, critical for the posterior microscope inspection.

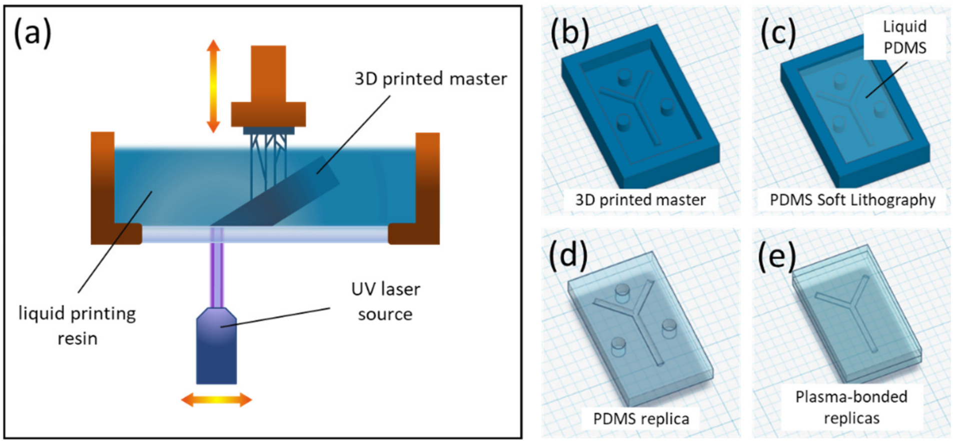

Supplementary Figure S1: (a) Stereolithography printer working principle. The master was designed in CAD (b) and 3D printed in resin (c). Soft lithography of PDMS of the master was performed to obtain two matching replicas of polymer (d) that are subsequently bonded to obtain a fully functional device (e).

Numerical methods

Fluid dynamics

Star-CCM+ software [Star-CCM+ documentation] was used to design geometries, build a grid and carry-on numerical simulations. Segregated Flow solver and finite volume method (FVM) were used in order to solve the fluid-dynamic equations (1), (2). Fluid dynamics has been simulated by incompressible Navier-Stokes equations described below:

$$\Delta\vec{v} = 0 \qquad (1)$$

$$\rho(\vec{v} \cdot \nabla)\vec{v} = -\nabla p + \mu\Delta\vec{v} \qquad (2)$$

where ρ is the fluid density, μ fluid viscosity, $\vec{v}$ fluid velocity and p pressure.

Computations were run until a steady state was reached and convergence monitors were set in 1e-5.

The domain used to perform the numerical simulation was a bifurcation with an opening angle α equal to 90º and sharp vertex.

Flow conditions

Medium has been considered as fluid in experiments and in numerical simulations with a density of 1025 kg/m^3 and a viscosity equal to 0.0015 Pa·s. Zero pressure was set at the outlets and the inlet boundary condition was defined in order to achieve the same Reynolds number as those reached in the experiments and, thus the same flow behavior. Reynolds number was always less than 100 and thus, laminar flow was guaranteed for all the simulations.

Particle properties

Rigid red blood cells (RBC) were modeled with biconcave shape (Supplementary Figure S2) with a diameter of 6 µm. The final geometry is obtained as follows: 1) the geometry with biconcave shape is designed using CAD techniques and 2) it is filled with 54 spheres. This structure formed by spheres allows us to simulate the RBC behavior in the flow.

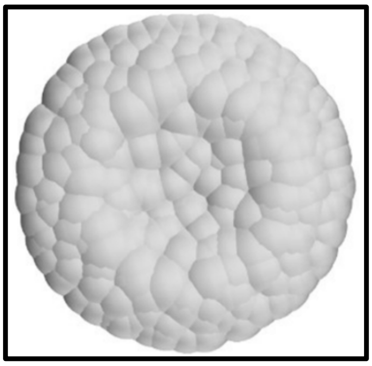

Supplementary Figure S2. Red blood cell formed by 54 spheres

The forces that act on the particles are drag and lift force and gravity as defined below [4]:

Drag Force

$F_d = \frac{1}{2} C_d \rho A_p |v_s| v_s$

(3)

where C_d is the drag coefficient of the particle, ρ is the density of the continuous phase, $v_s = v - v_s$ is the particle slip velocity with v being the instantaneous velocity of the continuous phase and A_p is the projected area of the particle. Schiller-Naumann was chosen as the method to define the drag coefficient because it is suitable for spherical solid particles. Since the Particle Reynolds number is always less than 1, the drag coefficient is defined as follows:

$C_d = \frac{24}{Re_p}(1 + 0.15 {Re_p}^{0.687})$

(4)

where:

$Re_p = \frac{\rho |v_s| D_p}{\mu}$

(5)

with D_p the particle diameter and μ the dynamic viscosity.

Gravity

$F_g = m_p * g$

(6)

with m_p the particle mass and g the gravitational acceleration vector.

Lift Force

Lift forces can arise from particle spin, particle shear or both. Particle spin lift force applies to a spinning particle moving relative to fluid. The force is given by:

$$F_{LR} = \frac{\rho\pi}{8} {D_p}^2 C_{LR} |v_s| \frac{\Omega \; x \; v_s}{|\Omega|} \tag{7}$$

where C_{LR} is the coefficient of rotational lift [5] and Ω is the angular velocity relative to the particle motion:

$$C_{LR} = 0.45 + \left(\frac{Re_R}{Re_p} - 0.45\right) e^{-0.5684 {Re_R}^{0.4} {Re_p}^{0.3}} \tag{8}$$

$$\Omega = \frac{1}{2} \nabla \; x \; v - \omega_p \tag{9}$$

where v is the fluid velocity and ω_p is the angular velocity of the particle.
The second lift force considered (shear lift force) applies to a particle moving relative to a fluid and it is defined as [6],

$$F_{LS} = C_{LS} \frac{\rho\pi}{8} {D_p}^3 (v_s \; x \; \omega) \tag{10}$$

where D_p is the particle diameter, v_s is the slip velocity, C_{LS} is the shear lift coefficient and ω is the curl of the fluid velocity. These coefficients are defined in the equations (11) y (12),

$$\omega = \nabla \; x \; v \tag{11}$$

$$C_{LS} = \frac{4.1126}{{Re_s}^{0.5}} \; f Re_p, Re_s \tag{12}$$

where

$$f Re_p, Re_s = \{(1 - 0.3314\beta^{0.5}) e^{-0.1 Re_p} + 0.3314\beta^{0.5}, (Re_p \leq 40) \; 0.0524 \, (\beta Re_p)^{0.5}, \qquad (Re_p > 40) \tag{13}$$

with

$$\beta = 0.5 \frac{Re_s}{Re_p} \tag{14}$$

where

$$Re_s = \frac{\rho {D_p}^2 |\omega|}{\mu} \tag{15}$$

Analysis of mesh independence

Numeric mesh was built using polyhedral mesh refined near the wall with hexahedral layers to improve calculation accuracy. In order to ensure that the results obtained do not depend on the

discretization of the geometry, the value of maximum velocity was studied for 4 different types of mesh in every geometry. All the simulations were repeated for the four different grids. In Table 1, the specifications of each grid are summarized for the geometry. The maximum value of the velocity of the fluid flow was chosen as the control variable for its significance in the problems analyzed. The circulating fluid considered for this test is the same that those used in the experiments (medium). Note that the variations in the magnitude 'Maximum velocity' were always less than 5%. For this reason, Mesh 1 was chosen for the simulations presented in the main text as they required less computational effort.

Table 1: Specifications of each grid for the geometry

	**Cells**	**Max. Velocity magnitude (m/s)**	**Variation**
**Mesh 1**	18505	0.029944	**-------**
**Mesh 2**	66927	0.030382	1.44 %
**Mesh 3**	114005	0.030930	3.19 %
**Mesh 4**	301415	0.030969	3.31 %

Results

Supplementary Figures

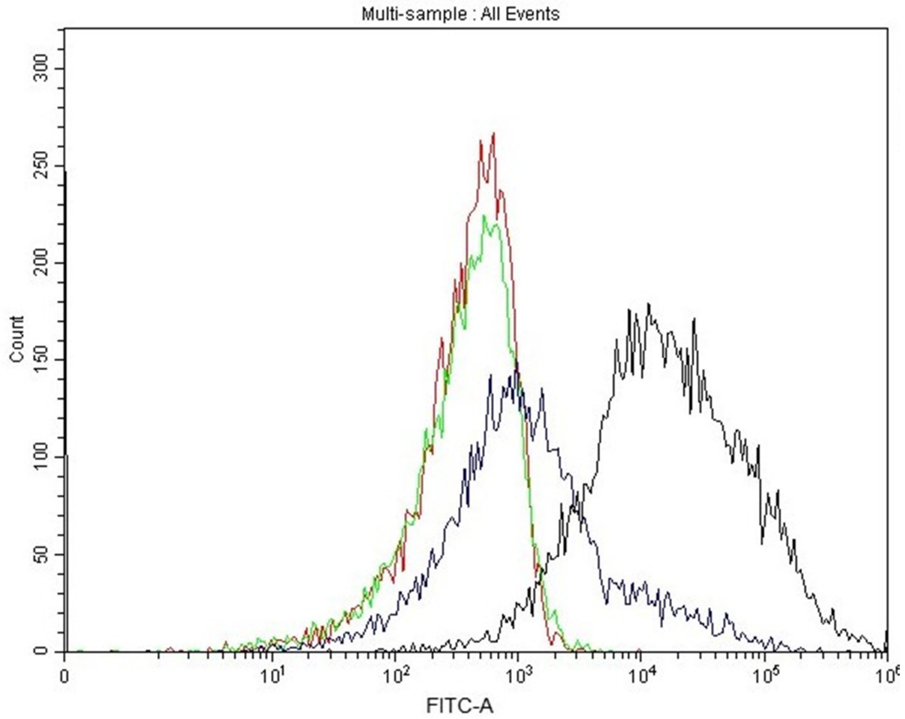

Figure S3: Glucose induced conformational change of CD47 in erythrocytes. Representative histograms of FITC-labeled CD47 (clone 2D3) for fresh (G0, red), G5 (green), G50 (blue) and G100 (black) erythrocytes obtained by flow cytometry. Anti-CD47 antibodies detect conformational dependent (clone 2D3) epitopes of CD47.

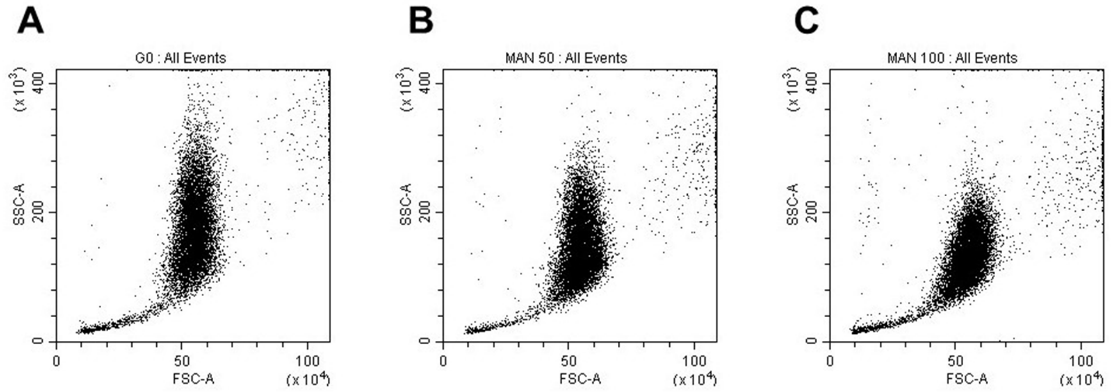

Figure S4: Mannitol induced hyperosmolarity did not affect erythrocyte integrity. Erythrocytes morphology in A) absence or in presence of mannitol (B) 50 mM and C) 100 mM) was investigated by flow cytometry

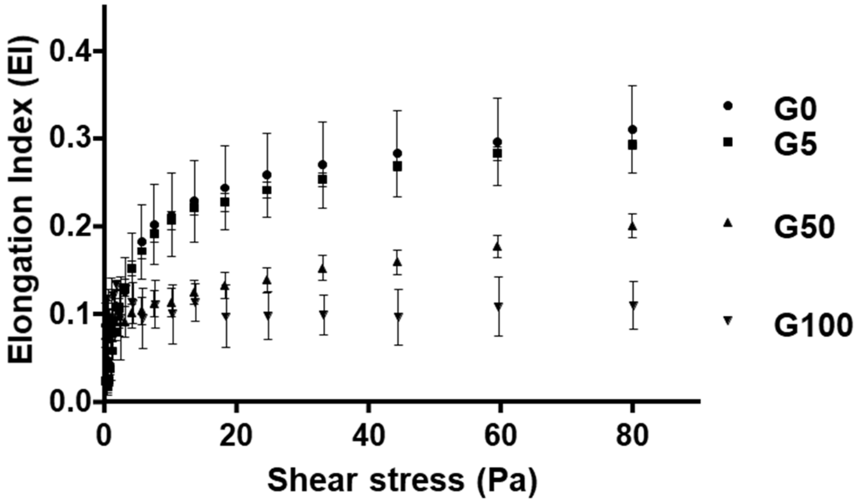

Figure S5: Glucose induced impairment of Erythrocytes deformability. Curves represent erythrocyte deformation as elongation index as a function of shear stress for fresh (-●-); G5 (-■-); G50 (-▲-) and G100 (-▼-);) erythrocyte samples. Data are means ± SEM of three independent experiments.

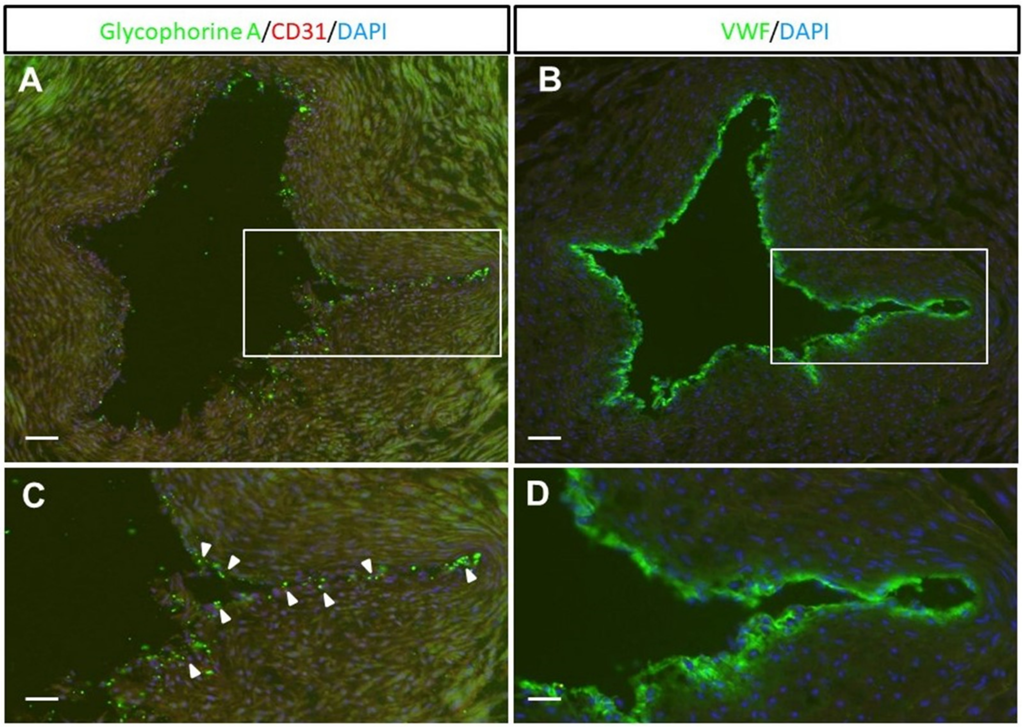

Figure S6: Glycophorin A/ CD31 and von Willebrand factor (vWF) immunostaining in human artery sections from umbilical cord incubated with glycated erythrocytes (G50). Arteries from human umbilical cords were incubated with G50 and were analyzed for the presence of glycophorin A (A & C) and vWF (B & D). Interactions between luminal endothelial cells and glycated erythrocytes are observed in G50-incubated artery, with a marked glycophorin A immunostaining in endothelial lining (see arrows). High magnification images of the areas delimited by squares are presented in C & D for arteries incubated with G50. White bars represent 200 µm (A-B) and 40 µm (C-D).

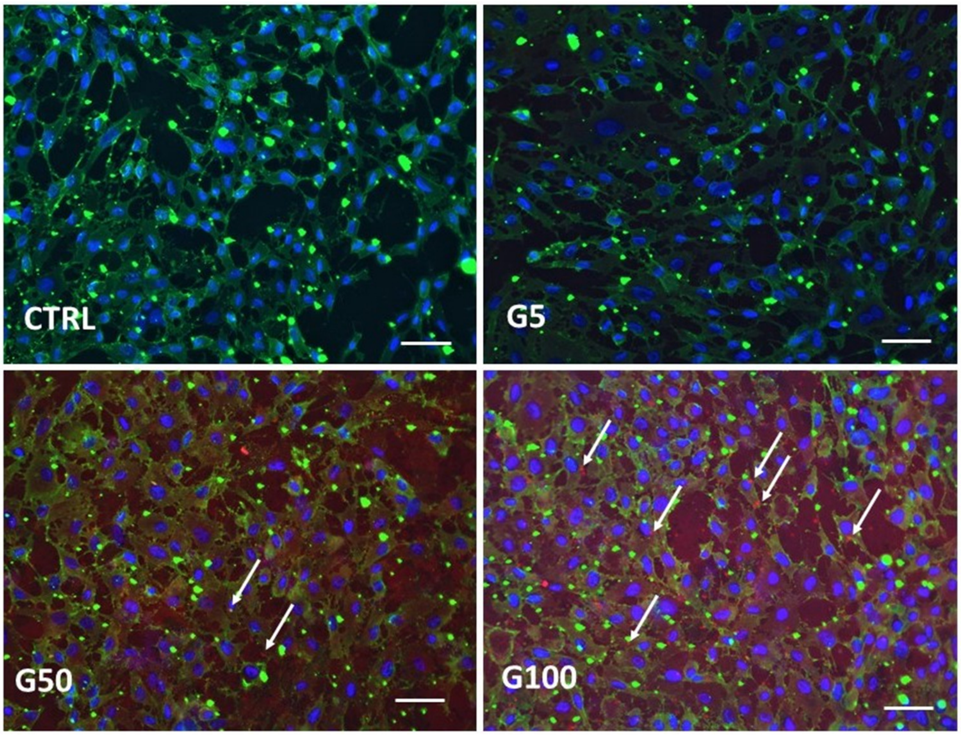

Figure S7: Erythrocyte glycation by glucose induced erythrophagocytosis in HUVEC cells.

Human endothelial cells HUVEC were incubated without or with pHrodo-labeled G5, G50 and G100 erythrocytes for 6 h at 37°C. After incubation, cells were washed three times with PBS 1X to discard unbound erythrocytes.

Epifluorescence imaging analysis after immunostaining of HUVEC (CD31 in green) and erythrocytes (pHrodo in red) after co-incubation without (CTRL) or with G5, G50 or G100 erythrocytes. Cell nuclei were stained with DAPI (blue). Scale bar = 40 μm.

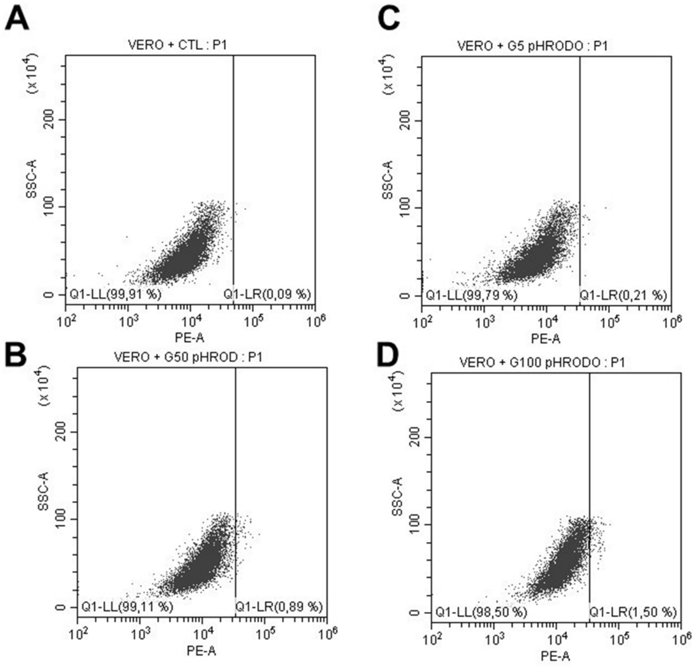

Figure S8: Erythrocyte glycation by glucose did not induce erythrophagocytosis in VERO cells.

Erythrophagocytosis was investigated by FACS using pHrodo fluorescent probe. A-D) Typical representative FACS dot plots after VERO cells stimulation for 12 hours without A) or with pH rodo labeled B) G5, C) G50, D) G100 erythrocytes.

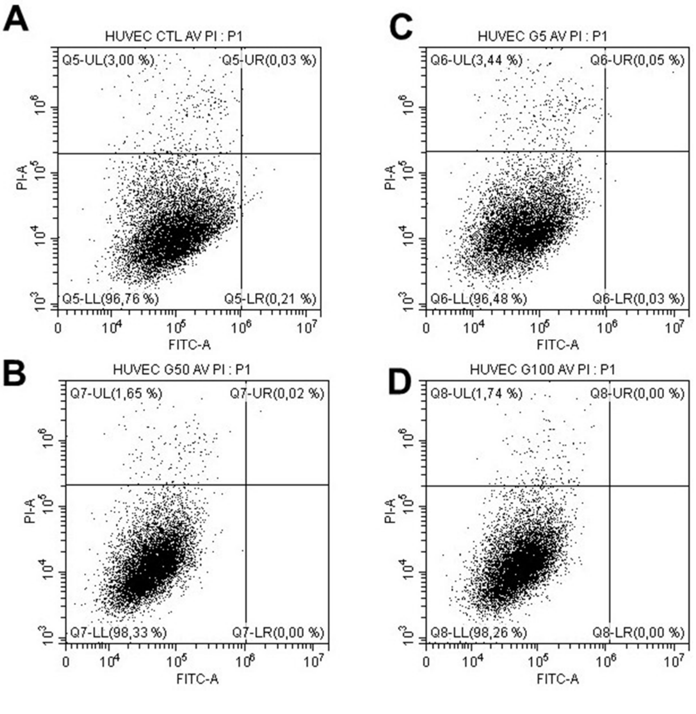

Figure S9: Erythrophagocytosis did not impact HUVEC cells viability.

Apoptosis/necrosis were investigated by FACS using FITC labeled Annexin V and propidium iodide fluorescent probe. A-D) Typical representative FACS dot plots after HUVEC cells stimulation for 6 hours without A) or with B) G5, C) G50, D) G100 erythrocytes.

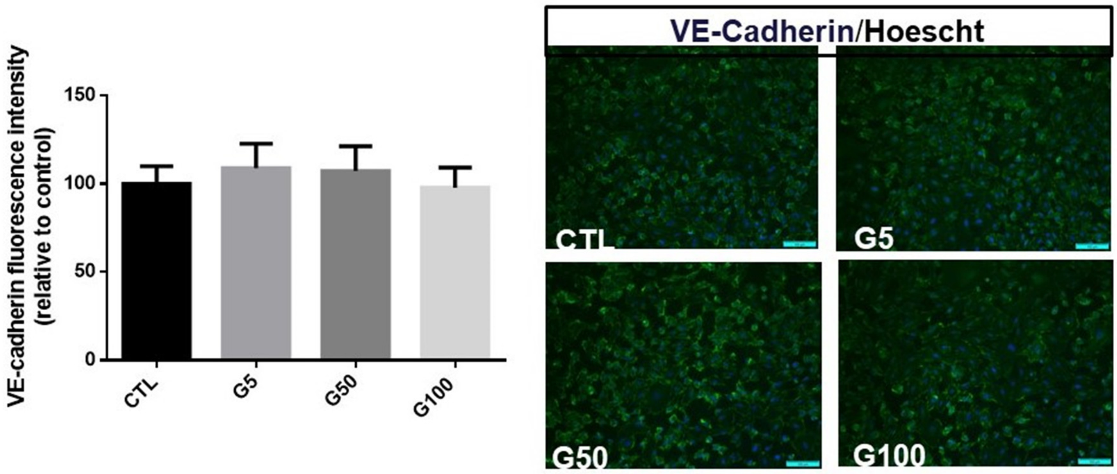

Figure S10: Erythrophagocytosis did not modulate VE-cadherin expression in HUVEC cells.

Human endothelial cells HUVEC were incubated without or with G5, G50 and G100 erythrocytes for 6 h at 37°C and were analyzed for the presence of VE-cadherin (green). Cell nuclei were stained with Hoescht (blue). Scale bar = 40 μm.

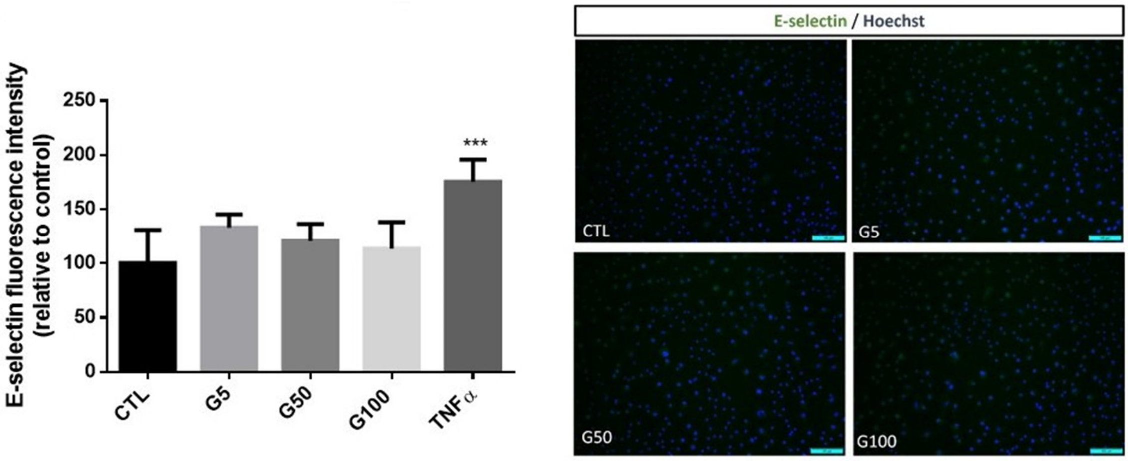

Figure S11: Erythrophagocytosis did not modulate E-selectin expression in HUVEC cells.

Human endothelial cells HUVEC were incubated without or with G5, G50 and G100 erythrocytes for 6 h at 37°C and were analyzed for the presence of E-selectin (green). TNFa (?? μg/μL) stimulation was used as positive control. Cell nuclei were stained with Hoescht (blue). Scale bar = 40 μm.

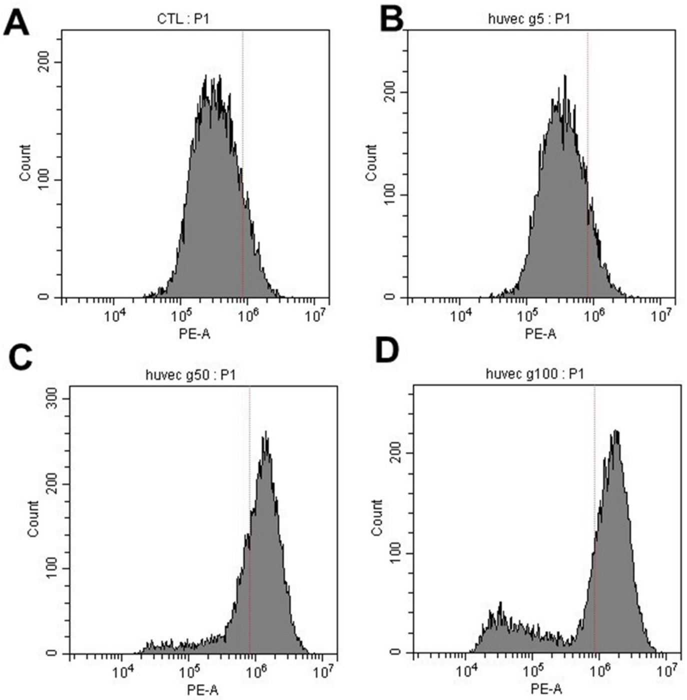

Figure S12: Induction of mitochondrial oxidative stress by G50 and G100 erythrocytes.
Mitochondrial superoxide production was evaluated in cells using MitoSOX Red fluorescent dye at 1 h treatment with control, G5, G50 and G100 erythrocytes. (A-D) Representative histograms of MitoSOX fluorescence were obtained by FACS.

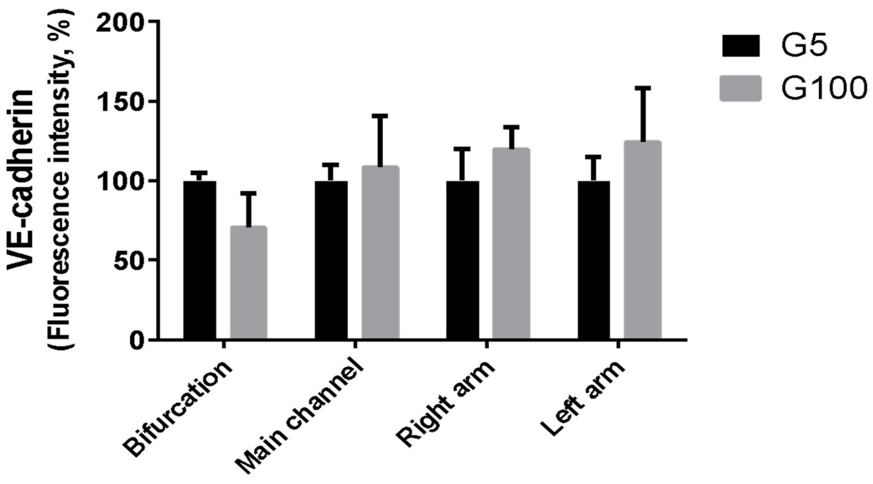

Figure S13: VE-Cadherin expression in HUVEC after flow experiments with glycated or non-glycated erythrocytes.
VE-cadherin expression measured by immunocytochemistry in HUVEC at the fluidic device after the flow assay with glycated (G100) and non-glycated (G5) erythrocytes during 5 h at a flow rate of 3 ml/min. Data were obtained from image analysis at 4 different points on each channel: main channel, bifurcation, right and left arms. Columns represent mean ± s.e.m. (in vertical bars) expressed as percentage of fluorescence intensity measured at the bifurcation of non-glycated erythrocytes experiment. At least three independent experiments were made for each condition and no statistical significant changes were observed between any measurement.

Numerical simulations identify the vulnerable zones at the bifurcation

A video of the numerical simulation created to reproduce the flow of erythrocytes through the fluidic device created is shown in Supplementary Video S1. The movement of the erythrocytes are recreated by the simulation.

In Supplementary Video S2, zoom views of the bifurcation and of the main straight portions of the channels are shown. The slower velocity of the erythrocytes near the outer walls in comparison with the zone near the carina is appreciated in the bifurcation portion. This difference is not appreciated in the straight portion of the main channel.

References

1. Carnero, B.; Bao-Varela, C.; Gómez-Varela, A.I.; Álvarez, E.; Flores-Arias, M.T. Microfluidic devices manufacturing with a stereolithographic printer for biological applications. *Mater. Sci. Eng. C* **2021**, *129*, 112388.
2. McDonald, J.C.; Duffy, D.C.; Anderson, J.R.; Chiu, D.T.; Wu, H.; Schueller, O.J.; Whitesides, G.M. Fabrication of microfluidic systems in poly(dimethylsiloxane). *Electrophoresis* **2000**, *21*, 27–40. https://doi.org/10.1002/(SICI)1522-2683(20000101)21:1<27::AID-ELPS27>3.0.CO;2-C.
3. Fuard, D.; Tzvetkova-Chevolleau, T.; Decossas, S.; Tracqui, P.; Schiavone, P. Optimization of poly-di-methyl-siloxane (PDMS) substrates for studying cellular adhesion and motility. *Microelectron. Eng.* **2008**, *85*, 1289–1293.
4. Ferziger, J.H.; Perić, M. *Computational Methods for Fluid Dynamics*; Springer: Berlin/Heidelberg, Germany, 2002. https://doi.org/10.1007/978-3-642 56026-.2
5. Sommerfeld, M. *Theoretical and Experimental Modeling of Particulate Flows*; Lecture Series; The von Karman Institute for Fluid Dynamics: Sint-Genesius-Rode, Belgium, 2000.
6. Saffman, P.G.T. The lift on a small sphere in a slow shear flow. *J. Fluid Mech.* **1965**, *22*, 385–400.
