# Supplementary figures and images for: Impact of Enhanced Phagocytosis of Glycated Erythrocytes on Human Endothelial Cell Functions
Source: Cells. 2022 Jul 14;11(14):2200. doi: 10.3390/cells11142200 (PMC9351689; doi:10.3390/cells11142200)

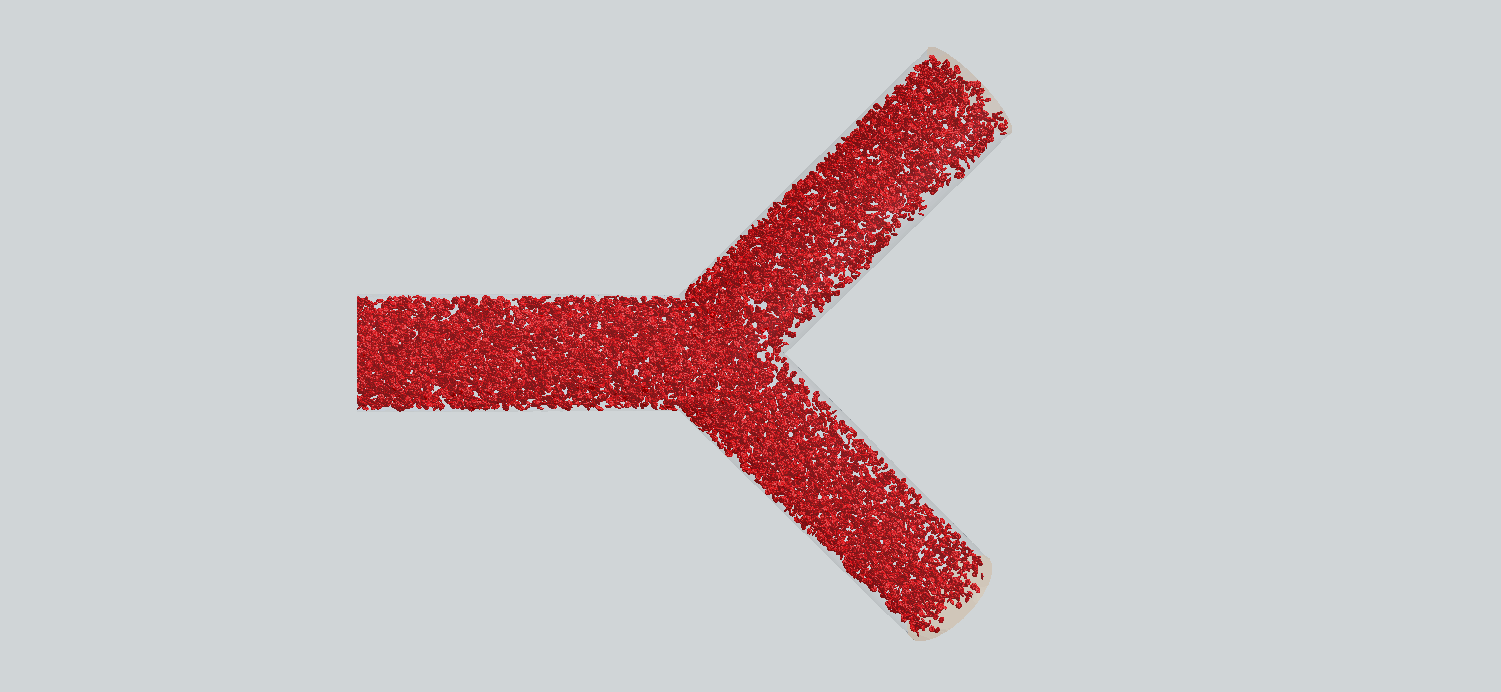

Supplement: Supplementary file 1 [file cells-11-02200-s001.zip › 03a_Supplementary_Video_1.gif]

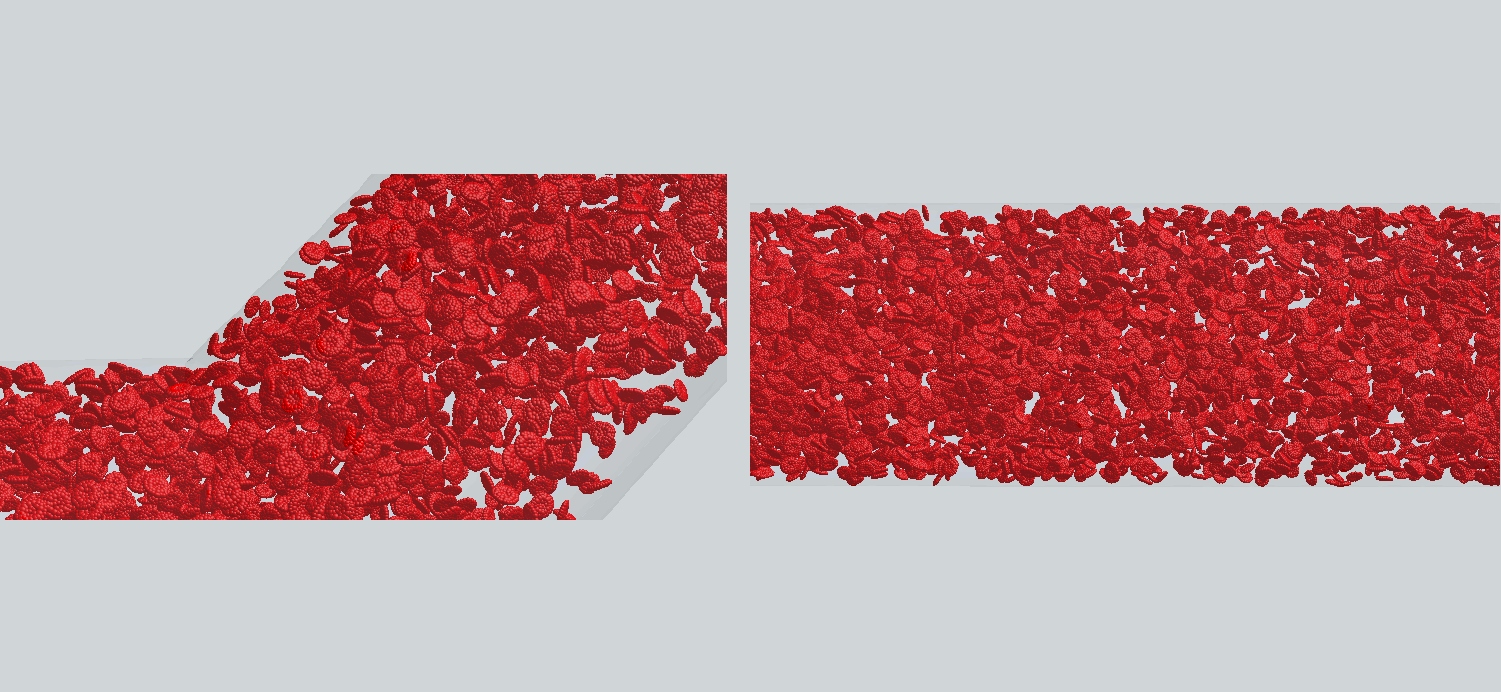

Supplement: Supplementary file 1 [file cells-11-02200-s001.zip › 03b_Supplementary_Video_2_Zoom.gif]
